# Supplementary material for: Examination of food consumption in United States adults and the prevalence of inflammatory bowel disease using National Health Interview Survey 2015
Source: PLoS One. 2020 Apr 23;15(4):e0232157. doi: 10.1371/journal.pone.0232157 (PMC7179926; doi:10.1371/journal.pone.0232157)
Supplement: S1 Table — (DOCX) [file pone.0232157.s001.docx]

| **Supplemental Table 1.1 Survey participant demography characteristics, NHIS 2015^a^** | | | | |  |
| --- | --- | --- | --- | --- | --- |
|  |  | **Unweighted, Unadjusted** | | |  |
| **Demographic Characteristics (N=Sample total)** | **Sub-categories** | **N (Total)** | **Percent** | **95 % CI** |  |
| Sample population |  | 103,789 |  |  |  |
|  | Sample adults (≥ 18 yrs old) | 33,672 | 32.44 | (32.16 - 32.73) |  |
| Gender | Men | 15,071 | 44.76 | (44.23 - 45.29) |  |
| (N=33,672) | Women | 18,601 | 55.24 | (54.71 - 55.77) |  |
| Age categories (yrs) | 18-24 | 2,890 | 8.58 | (8.29 - 8.89) |  |
| (N=33,672) | 25-34 | 5,783 | 17.17 | (16.78 - 17.58) |  |
|  | 35-44 | 5,284 | 15.69 | (15.31 - 16.09) |  |
|  | 45-54 | 5,566 | 16.53 | (16.14 - 16.93) |  |
|  | 55-64 | 5,771 | 17.14 | (16.74 - 17.55) |  |
|  | 65-74 | 4,695 | 13.94 | (13.58 - 14.32) |  |
|  | 75-85 | 3,683 | 10.94 | (10.61 - 11.28) |  |
| Ethnicity | Hispanic | 5,591 | 16.6 | (16.21 - 17.01) |  |
| (N=33,672) | Non-Hispanic | 28,081 | 83.4 | (82.99 - 83.79) |  |
| Race | White | 25,831 | 76.71 | (76.26 - 77.16) |  |
| (N=33,672) | Black or African-American | 4,673 | 13.88 | (13.51 - 14.25) |  |
|  | American Indian or Alaska Native | 392 | 1.16 | (1.06 - 1.28) |  |
|  | Asian | 1,983 | 5.89 | (5.64 - 6.15) |  |
|  | Multiple race | 699 | 2.08 | (1.93 - 2.23) |  |
|  | Unknown | 94 | 0.28 | (0.23 - 0.34) |  |
| Region | Northeast | 5,580 | 16.57 | (16.18 - 16.97) |  |
| (N=33,672) | Midwest | 7,102 | 21.09 | (20.66 - 21.53) |  |
|  | South | 11,646 | 34.59 | (34.08 - 35.1) |  |
|  | West | 9,344 | 27.75 | (27.27 - 28.23) |  |
| Highest education level completed | K or never attended | 134 | 0.40 | (0.34 - 0.47) |  |
| (N=33,672) | Primary school only | 457 | 1.36 | (1.24 - 1.49) |  |
|  | Junior HS only | 1223 | 3.63 | (3.44 - 3.84) |  |
|  | Some HS (Did not graduate) | 2,867 | 8.51 | (8.22 - 8.82) |  |
|  | HS graduate or GED | 8,359 | 24.82 | (24.37 - 25.29) |  |
|  | Some college (Did not graduate) | 6,570 | 19.51 | (19.09 - 19.94) |  |
|  | 2-year college | 3,866 | 11.48 | (11.15 - 11.83) |  |
|  | 4-year college | 6,224 | 18.48 | (18.07 - 18.9) |  |
|  | Advanced and Terminal Degrees | 3,824 | 11.36 | (11.02 - 11.7) |  |
|  | Unknown | 148 | 0.44 | (0.37 - 0.52) |  |
| Poverty status^b^ | Poor | 5,341 | 15.86 | (15.48 - 16.26) |  |
| (N=33,672) | Near poor | 7,020 | 20.85 | (20.42 - 21.29) |  |
|  | Not poor | 21,311 | 63.29 | (62.77 - 63.8) |  |
| Ever smoked^c^ | Yes | 16,853 | 50.05 | (49.52 - 50.58) |  |
| (N=33,672) | Never | 15,548 | 46.17 | (45.64 - 46.71) |  |
|  | Unknown | 1,271 | 3.77 | (3.58 - 3.98) |  |
| Alcohol user status^d^ | Abstainer | 6,930 | 20.58 | (20.15 - 21.02) |  |
| (N=33,672) | Former drinker | 5,252 | 15.6 | (15.21 - 15.99) |  |
|  | Current drinker | 21,053 | 62.52 | (62.01 - 63.04) |  |
|  | Unknown | 437 | 1.3 | (1.18 - 1.42) |  |
| Alcohol consumption status^e^ | Abstainer | 6,930 | 20.58 | (20.15 - 21.02) |  |
| (N=33,672) | Infrequent drinker | 7,584 | 22.52 | (22.08 - 22.97) |  |
|  | Regular or Light drinker | 11,998 | 35.63 | (35.12 - 36.15) |  |
|  | Moderate drinker | 4,936 | 14.66 | (14.29 - 15.04) |  |
|  | Heavy drinker | 1,679 | 4.99 | (4.76 - 5.22) |  |
|  | Unknown | 545 | 1.62 | (1.49 - 1.76) |  |
| Body Mass Index (BMI) | Underweight, (< 18.5) | 603 | 1.79 | (1.65 - 1.94) |  |
| (N=33,672) | Healthy (18.5 to < 25) | 10,863 | 32.26 | (31.76 - 32.76) |  |
|  | Overweight (25 to < 30) | 11,045 | 32.8 | (32.3 - 33.31) |  |
|  | Obese (30 and over) | 10,016 | 29.75 | (29.26 - 30.24) |  |
|  | Missing or Unascertained | 1,145 | 3.4 | (3.21 - 3.6) |  |
| IBD | Yes | 454 | 1.35 | (1.23 - 1.48) |  |
| (N=33,672) | No | 33,172 | 98.52 | (98.38 - 98.64) |  |
|  | Refused or Don't Know | 46 | 0.14 | (0.1 - 0.18) |  |
|  |  |  |  |  |  |
|  |  |  |  |  |  |
| ^a^Unweighted frequency and their percentages are reported; Data source: Person file, Sample Adult file, Imputed Income files, Sample Adult Cancer file from 2015 NHIS Data release source (https://www.cdc.gov/nchs/nhis/nhis_2015_data_release.htm) | | | | |  |
| ^b^Poverty status: Poor: < 100% of poverty threshold; Near Poor: 100-200% of poverty threshold; Not Poor: ≥ 200% of poverty threshold | | | | |  |
| ^c^Any participants who answered "Yes" to any of the following questions:1) Ever smoked a cigarette at least 100 times, in life time; 2) Ever smoked a cigar-related product, even once; 3) Ever smoked a pipe filled with tobacco, even once; 4) Ever used smokeless tobacco in entire life. Participant must answer "No" to all four questions to be considered as Never smoked. | | | | | |
| ^d^User status: Abstainer: < 12 times in lifetime; Former: > 12 times in lifetime but none in past year; Current: > 12 times in lifetime and consumed at least 1 drink in the past year | | | | | |
| ^e^Consumption status: Abstainer: < 12 times in lifetime; Infrequent: ≤ 12 time a year; Regular or Light: > 12 time a year but ≤ 3 a week in the past year; Moderate: 3-14 times a week for men or 3-7 times a week for women; Heavy: > 14 times a week for men or > 7 times a week for women | | | | | |
| K (Kindergarten); HS (High school); GED (General Education Development) | | |  |  |  |

| **Supplemental Table 1.2 Weighted demographic characteristics of US adults with IBD, NHIS 2015^a^** | | | | | | | |  |
| --- | --- | --- | --- | --- | --- | --- | --- | --- |
|  |  | **IBD = Yes**  **(Total N=3,087,297)** | | **IBD = No**  **(Total N=239,025,451)** | | **IBD =Yes vs IBD =No** | |  |
|  |  | **Weighted, Unadjusted** | | **Weighted, Unadjusted** | | **Adjusted Wald Test** | |  |
| **Demographic Characteristics** | **Sub-categories** | **Percent** | **95 % CI** | **Percent** | **95 % CI** | **F-test^c^** | **p-value** |  |
| Adult Population with IBD |  | 1.28 | (1.27-1.28) | 98.72 | (98.72-98.73) | - | - |  |
| Gender | Men | 42.59 | (42.53 - 42.64) | 48.27 | (48.27 - 48.28) |  |  |  |
|  | Women | 57.41 | (57.36 - 57.47) | 51.73 | (51.72 - 51.73) | 2.96 | 0.0866 |  |
| Age categories (yrs) | 18-24 | 4.95 | (4.92 - 4.97) | 12.43 | (12.43 - 12.43) | 23.95 | <0.0001* |  |
|  | 25-34 | 15.89 | (15.85 - 15.93) | 17.65 | (17.64 - 17.65) | 0.52 | 0.4730 |  |
|  | 35-44 | 12.13 | (12.1 - 12.17) | 16.65 | (16.65 - 16.66) | 4.56 | 0.0335* |  |
|  | 45-54 | 17.83 | (17.79 - 17.87) | 17.68 | (17.67 - 17.68) | 0.00 | 0.9498 |  |
|  | 55-64 | 23.13 | (23.08 - 23.18) | 16.53 | (16.52 - 16.53) | 6.45 | 0.0116* |  |
|  | 65-74 | 15.87 | (15.83 - 15.91) | 11.19 | (11.18 - 11.19) | 4.62 | 0.0324* |  |
|  | 75-85 | 10.19 | (10.16 - 10.23) | 7.88 | (7.87 - 7.88) | 1.58 | 0.2098 |  |
| Ethnicity | Hispanic | 12.80 | (12.76 - 12.84) | 15.62 | (15.62 - 15.63) | 1.84 | 0.1762 |  |
|  | Non-Hispanic | 87.20 | (87.16 - 87.24) | 84.38 | (84.37 - 84.38) | 1.84 | 0.1762 |  |
| Race | White | 88.18 | (88.15 - 88.22) | 78.95 | (78.94 - 78.95) | 21.67 | <0.0001* |  |
|  | Black or African-American | 5.63 | (5.60 - 5.66) | 12.41 | (12.41 - 12.42) | 29.28 | <0.0001* |  |
|  | American Indian or Alaska Native | 0.23 | (0.23 - 0.24) | 0.96 | (0.96 - 0.97) | 15.80 | 0.0001* |  |
|  | Asian | 3.76 | (3.74 - 3.78) | 5.94 | (5.94 - 5.95) | 2.54 | 0.1187 |  |
|  | Multiple race | 2.20 | (2.18 - 2.21) | 1.73 | (1.73 - 1.73) | 0.26 | 0.6123 |  |
| Region | Northeast | 19.35 | (19.30 - 19.39) | 17.41 | (17.40 - 17.41) | 0.46 | 0.5004 |  |
|  | Midwest | 22.09 | (22.05 - 22.14) | 22.41 | (22.41 - 22.42) | 0.01 | 0.9085 |  |
|  | South | 38.32 | (38.26 - 38.37) | 37.13 | (37.13 - 37.14) | 0.13 | 0.7159 |  |
|  | West | 20.25 | (20.20 - 20.29) | 23.04 | (23.04 - 23.05) | 1.25 | 0.2645 |  |
| Education attainment | K or never attended | 0.08 | (0.08 - 0.09) | 0.33 | (0.32 - 0.33) | 7.76 | 0.0057* |  |
|  | Primary school only | 0.97 | (0.95 - 0.98) | 1.07 | (1.06 - 1.07) | 0.02 | 0.8748 |  |
|  | Junior HS only | 3.66 | (3.64 - 3.68) | 2.94 | (2.93 - 2.94) | 0.31 | 0.5754 |  |
|  | Some HS (Did not graduate) | 12.02 | (11.99 - 12.06) | 8.20 | (8.19 - 8.2) | 2.47 | 0.1172 |  |
|  | HS graduate or GED | 25.06 | (25.01 - 25.11) | 24.80 | (24.79 - 24.8) | 0.02 | 0.8990 |  |
|  | Some college (Did not graduate) | 19.94 | (19.9 - 19.99) | 19.65 | (19.64 - 19.65) | 0.02 | 0.8769 |  |
|  | 2-year college | 10.71 | (10.68 - 10.75) | 11.52 | (11.51 - 11.52) | 0.16 | 0.6896 |  |
|  | 4-year college | 17.05 | (17.01 - 17.09) | 19.72 | (19.72 - 19.73) | 1.17 | 0.2814 |  |
|  | Advanced and Terminal Degrees | 10.51 | (10.47 - 10.54) | 11.79 | (11.79 - 11.8) | 0.38 | 0.5371 |  |
| Poverty status^b^ | Poor | 15.73 | (15.69 - 15.77) | 12.18 | (12.18 - 12.19) | 2.98 | 0.0853 |  |
|  | Near poor | 17.95 | (17.91 - 18.00) | 18.67 | (18.66 - 18.67) | 0.08 | 0.7814 |  |
|  | Not poor | 66.32 | (66.26 - 66.37) | 69.15 | (69.15 - 69.16) | 0.91 | 0.3405 |  |
| Ever smoked^b^ | Yes | 57.15 | (57.09 - 57.2) | 50.88 | (50.87 - 50.89) |  |  |  |
|  | Never | 42.85 | (42.80 - 42.91) | 49.12 | (49.11 - 49.13) | 4.14 | 0.0427* |  |
| Alcohol user status^c^ | Abstainer | 16.16 | (16.12 - 16.21) | 20.69 | (20.68 - 20.69) | 3.45 | 0.0641 |  |
|  | Former drinker | 25.68 | (25.63 - 25.73) | 14.06 | (14.06 - 14.07) | 17.78 | <0.0001* |  |
|  | Current drinker | 58.15 | (58.1 - 58.21) | 65.75 | (65.24 - 65.25) | 5.18 | 0.0236* |  |
| Alcohol consumption status^d^ | Abstainer | 16.30 | (16.26 - 16.35) | 20.76 | (20.75 - 20.76) | 3.31 | 0.0700 |  |
|  | Infrequent drinker | 29.85 | (29.79 - 29.9) | 21.90 | (21.9 - 21.91) | 7.63 | 0.0061* |  |
|  | Regular or Light drinker | 36.81 | (36.75 - 36.86) | 36.64 | (36.63 - 36.64) | 0.00 | 0.9523 |  |
|  | Moderate drinker | 13.46 | (13.42 - 13.5) | 15.73 | (15.72 - 15.73) | 0.89 | 0.3450 |  |
|  | Heavy drinker | 3.59 | (3.57 - 3.61) | 4.98 | (4.98 - 4.98) | 1.71 | 0.1917 |  |
| Body Mass Index (kg/m^2^) | Underweight, (< 18.5) | 2.76 | (2.74 - 2.78) | 1.89 | (1.89 - 1.89) | 0.73 | 0.3925 |  |
|  | Healthy (18.5 to < 25) | 34.06 | (34.01 - 34.12) | 34.34 | (34.33 - 34.34) | 0.01 | 0.9296 |  |
|  | Overweight (25 to < 30) | 36.90 | (36.84 - 36.95) | 33.73 | (33.72 - 33.73) | 1.20 | 0.2738 |  |
|  | Obese (30 and over) | 26.27 | (26.22 - 26.32) | 30.05 | (30.04 - 30.06) | 1.76 | 0.1853 |  |
|  |  |  |  |  |  |  |  |  |
|  |  |  |  |  |  |  |  |  |
|  |  |  |  |  |  |  |  |  |
| ^a^Weighted frequency and their percentages (95% Confidence Interval) are reported; Data source: Person file, Sample Adult file, Imputed Income files, Sample Adult Cancer file from 2015 NHIS Data release source (https://www.cdc.gov/nchs/nhis/nhis_2015_data_release.htm). Weighting and Sampling units are based on population reported on 2010 US Census. | | | | | | | |  |
| ^b^Poverty status: Poor: Less than 100% of poverty threshold; Near Poor: 100% to less than 200% of poverty threshold; Not Poor: 200% of poverty threshold or greater | | | | | | | |  |
| ^b^Any participants who answered "Yes" to any of the following questions:1) Ever smoked a cigarette at least 100 times, in life time; 2) Ever smoked a cigar-related product, even once; 3) Ever smoked a pipe filled with tobacco, even once; 4) Ever used smokeless tobacco in entire life. Participant must answer "No" to all four questions to be considered Never smoked. | | | | | | | | |
| ^c^User status: Abstainer: Less than 12 times in lifetime; Former: > 12 in lifetime but none in past year; Current: > 12 in lifetime and consumed at least 1 drink in the past year. | | | | | | | | |
| ^d^Consumption status: Abstainer: Less than 12 times in lifetime; Infrequent: ≤ 12 time a year; Regular or Light: > 12 time a year but ≤ 3 a week in the past year; Moderate: 3-14 times a week for males or 3-7 times a week for females; Heavy: > 14 times a week for men or > 7 times a week for women. | | | | | | | | |
| ^e^Test of proportion (Adjusted Wald Test) was performed on the response subcategories (between IBD = Yes vs IBD=No). Unknown, Missing, or Unascertained are not included in the calculation of percentage and/or the test of proportions | | | | | | | | |
| K (Kindergarten); HS (High school); GED (General Education Development) | | | | | | | | |
| *Statistically significant, below the significance level of 0.05 | | | | | | | | |
