# Supplementary material for: Examination of food consumption in United States adults and the prevalence of inflammatory bowel disease using National Health Interview Survey 2015
Source: PLoS One. 2020 Apr 23;15(4):e0232157. doi: 10.1371/journal.pone.0232157 (PMC7179926; doi:10.1371/journal.pone.0232157)
Supplement: S2 Table — (DOCX) [file pone.0232157.s002.docx]

| **Supplemental Table 2 Food items listed in the Diet and Nutrition questionnaire from Cancer Control Supplement, NHIS^a^** | |
| --- | --- |
| **Food items^a^** | **Survey Question: "During the past month, how often did you eat/have [food item]? You can tell me per day, per week, or per month."** |
| Cereal | HOT or COLD cereals |
| Milk | MILK; Do not include soy milk or small amounts of milk in coffee or tea |
| Soda or pop | REGULAR SODA or pop that contains sugar |
| Fruit juice | 100% PURE fruit juice such as orange, mango, apple, grape and pineapple juices; Do not include fruit flavored drinks with added sugar or fruit juice you made at home |
| Coffee | COFFEE or tea that had sugar or honey added to it; Include coffee and tea you sweetened yourself and presweetened tea and coffee drinks such as Arizona Iced tea and Frappuccino. Do not include artificially sweetened coffee or diet tea. |
| Sport and Energy drink | SPORTS and ENERGY drinks such as Gatorade, Red Bull, and Vitamin water |
| Fruit drink | SWEETENED FRUIT DRINKS, such as Kool-aid, cranberry and lemonade; Include fruit drinks you made at home and added sugar to. |
| Fruit | FRUITS; Include fresh, frozen, or canned fruit. Do not include juices. |
| Salad | green leafy or lettuce SALAD, with or without other vegetables |
| Fries | any kind of FRIED POTATOES, including French fries, home fries, or hash brown potatoes |
| Potato | any OTHER KIND OF POTATOES, such as baked, boiled, mashed potatoes, sweet potatoes, or potato salad |
| Beans | refried beans, baked beans, beans in soup, pork and beans or any other type of cooked dried beans; Do not include green beans. |
| Brown rice | BROWN RICE or other cooked whole grains, such as bulgur, cracked wheat, or millet; Do not include white rice. |
| Vegetables | OTHER VEGETABLES (exclude lettuce salads, potatoes, cooked dried beans from previous question) |
| Salsa | Mexican-type SALSA made with tomato |
| Pizza | PIZZA; Include frozen pizza, fast food pizza, and homemade pizza |
| Tomato sauce | TOMATO SAUCES such as with spaghetti or noodles or mixed into foods such as lasagna; Do not include tomato sauce on pizza. |
| Cheese | CHEESE; Include cheese as a snack, cheese on burgers, sandwiches, and cheese in foods such as lasagna, quesadillas, or casseroles. Do not include cheese on pizza. |
| Red meat | RED MEAT, such as beef, pork, ham, or sausage; Do not include chicken, turkey, or seafood. |
| Processed meat | PROCESSED MEAT; such as bacon, lunch meats, or hot dogs |
| Whole grain bread | WHOLE GRAIN BREAD including toast, rolls and in sandwiches; Whole grain breads include whole wheat, rye, oatmeal and pumpernickel. Do not include white bread. |
| Candy | Chocolate, or any other types of CANDY; Do not include sugar-free candy. |
| Donut | DOUGHNUTS, sweet rolls, Danish, muffins, (pan dulce) or pop-tarts; Do not include sugar-free items. |
| Cookie | COOKIES, CAKE, PIE, or BROWNIES; Do not include sugar-free kinds. |
| Ice cream | ICE CREAM OR OTHER FROZEN DESSERTS; Do not include sugar-free kinds. |
| Popcorn | POPCORN |
|  |  |
| ^a^ Food items from Sample Adult Cancer file, Cancer Control Supplement module, National Health Interview and Survey 2015. https://www.cdc.gov/nchs/nhis/nhis_2015_data_release.htm | |
