# Supplementary material for: Examination of food consumption in United States adults and the prevalence of inflammatory bowel disease using National Health Interview Survey 2015
Source: PLoS One. 2020 Apr 23;15(4):e0232157. doi: 10.1371/journal.pone.0232157 (PMC7179926; doi:10.1371/journal.pone.0232157)
Supplement: S3 Table — (DOCX) [file pone.0232157.s003.docx]

| **Supplemental Table 3 Number of adult survey participants and weighted estimated population consuming listed food items from Cancer Control Supplement, NHIS 2015^a,b^** | | | | | | |
| --- | --- | --- | --- | --- | --- | --- |
|  |  |  | Unweighted, Unadjusted | | Weighted, Unadjusted | |
|  |  |  | **Sample population** | | **Estimated population** | |
| Food groups^c^ | Food items |  | N | Percent | N | Percent |
| Whole wheat grains | Popcorn |  | 16,018 | 50.61 | 115,866,849 | 51.10 |
|  | Cereal (hot or cold)^f^ |  | 22,855 | 71.80 | 163,222,824 | 71.56 |
|  | Brown rice |  | 15,372 | 48.48 | 113,758,180 | 50.08 |
|  | Whole grain bread |  | 24,147 | 76.32 | 174,334,319 | 76.89 |
| Fruits and vegetables | Fries |  | 24,663 | 77.71 | 181,717,511 | 79.76 |
|  | Salad (green leafy, lettuce) |  | 28,461 | 89.58 | 205,419,323 | 90.22 |
|  | Fruit juices (100% pure fruit juice) |  | 20,989 | 66.08 | 150,914,877 | 66.30 |
|  | Vegetables^d^ |  | 30,207 | 95.33 | 216,930,088 | 95.53 |
|  | Potato (non-fried) |  | 26,837 | 84.66 | 193,387,286 | 85.12 |
|  | Pizza (frozen, fast food, homemade)^f^ |  | 25,366 | 79.96 | 187,697,450 | 82.58 |
|  | Fruits (fresh, frozen, canned) |  | 29,680 | 93.43 | 212,278,941 | 93.24 |
|  | Tomato sauce |  | 25,831 | 81.59 | 188,308,837 | 83.03 |
|  | Salsa (made with tomatoes) |  | 20,018 | 63.16 | 147,990,000 | 65.19 |
|  | Beans |  | 24,606 | 77.62 | 176,203,909 | 77.61 |
| Dairy | Milk (cow milk, any type) |  | 24,041 | 75.59 | 172,634,448 | 75.75 |
|  | Cheese (excludes cheese on pizza) |  | 29,093 | 91.87 | 209,693,652 | 92.41 |
|  | Pizza (frozen, fast food, homemade)^f^ |  | 25,366 | 79.96 | 187,697,450 | 82.58 |
|  | Ice cream (frozen desserts)^f^ |  | 22,258 | 70.33 | 162,222,316 | 71.56 |
| Meat | Processed meat |  | 23,820 | 75.22 | 173,062,848 | 76.27 |
|  | Red meat |  | 28,814 | 90.98 | 207,146,299 | 91.29 |
| Sweetened food/drinks^e^ | Cereal (hot or cold)^f^ |  | 22,855 | 71.80 | 163,222,824 | 71.56 |
|  | Cookies (i.e. cake, pies, brownies) |  | 23,341 | 73.78 | 170,612,848 | 75.27 |
|  | Donut (i.e. Danish, pastries, muffins) |  | 17,336 | 54.76 | 127,529,051 | 56.23 |
|  | Coffee or tea (sugar or honey added) |  | 17,179 | 54.04 | 124,517,306 | 54.66 |
|  | Fruit drinks (sweetened with sugar) |  | 8,833 | 27.79 | 63,737,542 | 27.99 |
|  | Candy (i.e. chocolates) |  | 23,855 | 75.38 | 172,588,758 | 76.13 |
|  | Sports and energy drinks |  | 8,289 | 26.06 | 64,492,081 | 28.30 |
|  | Regular soda or pop |  | 17,403 | 54.71 | 127,613,230 | 55.98 |
|  | Ice cream (frozen desserts)^f^ |  | 22,258 | 70.33 | 162,222,316 | 71.56 |
|  |  |  |  |  |  |  |
|  |  |  |  |  |  |  |
|  |  |  |  |  |  |  |
|  |  |  |  |  |  |  |
|  |  |  |  |  |  |  |
|  |  |  |  |  |  |  |
|  |  |  |  |  |  |  |
| ^a^Weighted using sample weight [wtfa_sa]; Data source: Sample Adult Cancer file from 2015 NHIS Data release source (https://www.cdc.gov/nchs/nhis/nhis_2015_data_release.htm) | | | | | | |
| ^b^Additional details in survey questions can be found in NHIS 2015 Data release website: ftp://ftp.cdc.gov/pub/Health_Statistics/NCHS/Dataset_Documentation/NHIS/2015/cancerxx_layout.pdf | | | | | | |
| ^c^Food groups are based on the relationship previously established according the dietary guidelines. Details can be found on https://epi.grants.cancer.gov/nhanes/dietscreen/relationship.html. | | | | | | |
| ^d^Vegetables other than lettuce salads, potatoes, cooked beans in which participant already answered to in previous questions. | | | | | | |
| ^e^Food items in this group excludes artificially sweetened or sugar-free kinds | | | | | | |
| ^f^Food items appear in more than one food groups: Pizza, Ice cream, Cereal | | | | | | |
