# Supplementary material for: Examination of food consumption in United States adults and the prevalence of inflammatory bowel disease using National Health Interview Survey 2015
Source: PLoS One. 2020 Apr 23;15(4):e0232157. doi: 10.1371/journal.pone.0232157 (PMC7179926; doi:10.1371/journal.pone.0232157)
Supplement: S4 Table — (DOCX) [file pone.0232157.s004.docx]

| **Supplemental Table 4 Association (OR^h^) of food consumption and IBD in estimated US population, NHIS 2015^a,b^** | | | | | | | | | | | | | |
| --- | --- | --- | --- | --- | --- | --- | --- | --- | --- | --- | --- | --- | --- |
|  |  | Weighted, unadjusted | | | Weighted, Adjusted for Demography^f^ | | | Weighted, Adjusted for Lifestyle^g^ | | | Weighted, Adjusted for Demography and Lifestyle^f,g^ | | |
| Food groups^c^ | Food items | OR | p-value | 95% CI | OR | p-value | 95% CI | OR | p-value | 95% CI | OR | p-value | 95% CI |
| Whole wheat grains | Popcorn | 0.77 | 0.053 | (0.5838 - 1.0000) | 0.78 | 0.072 | (0.5920 - 1.0220) | 0.78 | 0.068 | (0.5885 - 1.0190) | 0.79 | 0.089 | (0.5975 - 1.0374) |
|  | Cereal (hot or cold)^i^ | 1.22 | 0.246 | (0.8728 - 1.6950) | 1.15 | 0.393 | (0.8310 - 1.5950) | 1.21 | 0.249 | (0.8720 - 1.6870) | 1.15 | 0.402 | (0.8307 - 1.5861) |
|  | Brown rice | 0.88 | 0.337 | (0.6738 - 1.1450) | 0.94 | 0.688 | (0.7210 - 1.2400) | 0.87 | 0.318 | (0.6682 - 1.1400) | 0.93 | 0.624 | (0.7098 - 1.2285) |
|  | Whole grain bread | 0.87 | 0.283 | (0.6716 - 1.1240) | 0.90 | 0.443 | (0.6950 - 1.1720) | 0.88 | 0.343 | (0.6763 - 1.1460) | 0.91 | 0.474 | (0.6943 - 1.1851) |
| Fruits and vegetables | Fries | 1.41 | 0.032* | (1.0299 - 1.9290) | 1.63 | 0.003* | (1.1890 - 2.2450) | 1.40 | 0.036* | (1.0220 - 1.9237) | 1.63 | 0.003* | (1.1831 - 2.2375) |
|  | Salad (green leafy, lettuce) | 0.72 | 0.068 | (0.5078 - 1.0200) | 0.71 | 0.057 | (0.4930 - 1.0100) | 0.73 | 0.085 | (0.5109 - 1.0444) | 0.71 | 0.063 | (0.4907 - 1.0189) |
|  | Fruit juices (100% pure fruit juice) | 0.82 | 0.147 | (0.6230 - 1.0730) | 0.89 | 0.417 | (0.6778 - 1.1750) | 0.82 | 0.155 | (0.6191 - 1.0797) | 0.89 | 0.424 | (0.6733 - 1.1814) |
|  | Vegetables^d^ | 0.75 | 0.256 | (0.4527 - 1.2355) | 0.69 | 0.147 | (0.4160 - 1.1400) | 0.74 | 0.243 | (0.4474 - 1.2270) | 0.68 | 0.138 | (0.4106 - 1.1317) |
|  | Potato (non-fried) | 1.16 | 0.418 | (0.8080 - 1.6700) | 1.04 | 0.818 | (0.7256 - 1.5000) | 1.24 | 0.254 | (0.8575 - 1.7856) | 1.11 | 0.581 | (0.7698 - 1.5940) |
|  | Pizza (frozen, fast food, homemade)^i^ | 0.90 | 0.556 | (0.6262 - 1.2860) | 1.02 | 0.899 | (0.7088 - 1.4790) | 0.95 | 0.787 | (0.6610 - 1.3680) | 1.09 | 0.644 | (0.7539 - 1.5782) |
|  | Fruits (fresh, frozen, canned) | 0.86 | 0.481 | (0.5591 - 1.3150) | 0.84 | 0.418 | (0.5500 - 1.2820) | 0.91 | 0.697 | (0.5620 - 1.4712) | 0.88 | 0.608 | (0.5481 - 1.4225) |
|  | Tomato sauce | 0.92 | 0.626 | (0.6475 - 1.2990) | 0.93 | 0.670 | (0.6457 - 1.3250) | 0.92 | 0.661 | (0.6491 - 1.3160) | 0.93 | 0.682 | (0.6446 - 1.3331) |
|  | Salsa (made with tomatoes) | 0.94 | 0.676 | (0.7118 - 1.2470) | 1.11 | 0.510 | (0.8068 - 1.5380) | 0.97 | 0.822 | (0.7287 - 1.2859) | 1.14 | 0.442 | (0.8195 - 1.5754) |
|  | Beans | 0.95 | 0.729 | (0.6952 - 1.2900) | 0.93 | 0.656 | (0.6815 - 1.2700) | 0.96 | 0.804 | (0.6977 - 1.3219) | 0.93 | 0.679 | (0.6785 - 1.2878) |
| Dairy | Milk (cow milk, any type) | 0.79 | 0.122 | (0.5840 - 1.0640) | 0.80 | 0.134 | (0.5910 - 1.0730) | 0.80 | 0.139 | (0.5905 - 1.0768) | 0.81 | 0.162 | (0.5964 - 1.0910) |
|  | Cheese (excludes cheese on pizza) | 1.19 | 0.453 | (0.7494 - 1.9050) | 1.18 | 0.501 | (0.7290 - 1.9040) | 1.21 | 0.419 | (0.7596 - 1.9333) | 1.18 | 0.492 | (0.7328 - 1.9061) |
|  | Pizza (frozen, fast food, homemade)^i^ | 0.90 | 0.556 | (0.6262 - 1.2860) | 1.02 | 0.899 | (0.7088 - 1.4790) | 0.95 | 0.778 | (0.6601 - 1.3647) | 1.09 | 0.644 | (0.7539 - 1.5782) |
|  | Ice cream (frozen desserts)^i^ | 1.00 | 0.982 | (0.7570 - 1.3290) | 1.02 | 0.906 | (0.7670 - 1.3470) | 1.01 | 0.966 | (0.7536 - 1.3434) | 1.02 | 0.894 | (0.7649 - 1.3593) |
| Meat | Processed meat | 1.25 | 0.149 | (0.9230 - 1.6800) | 1.25 | 0.166 | (0.9119 - 1.7064) | 1.32 | 0.088 | (0.9597 - 1.8049) | 1.31 | 0.097 | (0.9516 - 1.8033) |
|  | Red meat | 1.11 | 0.612 | (0.7380 - 1.6700) | 1.12 | 0.585 | (0.7414 - 1.6970) | 1.16 | 0.488 | (0.7591 - 1.7787) | 1.17 | 0.476 | (0.7612 - 1.7914) |
| Sweetened food/drinks^e^ | Cereal (hot or cold)^i^ | 1.22 | 0.246 | (0.8728 - 1.6950) | 1.15 | 0.393 | (0.8310 - 1.5950) | 1.21 | 0.249 | (0.8720 - 1.6870) | 1.15 | 0.402 | (0.8307 - 1.5861) |
|  | Cookies (i.e. cake, pies, brownies) | 1.19 | 0.286 | (0.8640 - 1.6300) | 1.19 | 0.275 | (0.8690 - 1.6340) | 1.16 | 0.363 | (0.8403 - 1.6067) | 1.16 | 0.359 | (0.8432 - 1.5981) |
|  | Donut (i.e. Danish, pastries, muffins) | 1.11 | 0.439 | (0.8560 - 1.4280) | 1.15 | 0.269 | (0.8940 - 1.4900) | 1.07 | 0.634 | (0.8171 - 1.3925) | 1.12 | 0.413 | (0.8565 - 1.4569) |
|  | Coffee or tea (sugar or honey added) | 0.93 | 0.586 | (0.7220 - 1.2023) | 1.01 | 0.942 | (0.7800 - 1.3060) | 0.94 | 0.616 | (0.7268 - 1.2084) | 1.01 | 0.925 | (0.7837 - 1.3076) |
|  | Fruit drinks (sweetened with sugar) | 1.07 | 0.590 | (0.8357 - 1.3700) | 1.21 | 0.121 | (0.9496 - 1.5530) | 1.05 | 0.685 | (0.8177 - 1.3577) | 1.20 | 0.156 | (0.9318 - 1.5488) |
|  | Candy (i.e. chocolates) | 1.07 | 0.682 | (0.7759 - 1.4700) | 1.08 | 0.642 | (0.7839 - 1.4830) | 1.09 | 0.619 | (0.7806 - 1.5148) | 1.09 | 0.595 | (0.7866 - 1.5184) |
|  | Sports and energy drinks | 1.06 | 0.651 | (0.8200 - 1.3700) | 1.48 | 0.010* | (1.0990 - 1.9869) | 1.08 | 0.575 | (0.8259 - 1.4100) | 1.50 | 0.008* | (1.1159 - 2.0272) |
|  | Regular soda or pop | 0.98 | 0.890 | (0.7440 - 1.2900) | 1.13 | 0.418 | (0.8366 - 1.5344) | 0.98 | 0.896 | (0.7345 - 1.3099) | 1.14 | 0.410 | (0.8325 - 1.5655) |
|  | Ice cream (frozen desserts)^i^ | 1.00 | 0.982 | (0.7220 - 1.2170) | 1.02 | 0.906 | (0.7670 - 1.3470) | 1.01 | 0.966 | (0.7536 - 1.3434) | 1.02 | 0.894 | (0.7649 - 1.3593) |
|  |  |  |  |  |  |  |  |  |  |  |  |  |  |
|  |  |  |  |  |  |  |  |  |  |  |  |  |  |
| ^a^Weighted using sample weight [wtfa_sa]. Logistic regression with IBD as outcome; Data source: Sample Adult Cancer file from 2015 NHIS Data release (https://www.cdc.gov/nchs/nhis/nhis_2015_data_release.htm) | | | | | | | | | | | | | |
| ^b^Additional details in survey questions can be found in NHIS 2015 Data release website: ftp://ftp.cdc.gov/pub/Health_Statistics/NCHS/Dataset_Documentation/NHIS/2015/cancerxx_layout.pdf | | | | | | | | | | | | | |
| ^c^Food groups are based on the relationship previously established according the dietary guidelines. Details can be found on https://epi.grants.cancer.gov/nhanes/dietscreen/relationship.html. | | | | | | | | | | | | | |
| ^d^Vegetables other than lettuce salads, potatoes, cooked beans in which participant already answered to in previous questions. | | | | | | | | | | | | | |
| ^e^Food items in this group excludes artificially sweetened or sugar-free kinds | | | | | | | | | | | | | |
| ^f^Each food item adjusted for demographic factors: Age, race, poverty status, sex, ethnicity, region | | | | | | | | | | | | | |
| ^g^Each food item adjusted for lifestyle factors: Smoking, alcohol user status, alcohol consumption rate, BMI | | | | | | | | | | | | | |
| ^h^Odds Ratio: (Odds of having IBD in those consuming food item/Odds of having IBD in those never consumed the food item) | | | | | | | | | | | | | |
| ^i^Food items appear in more than one food groups: Pizza, Ice cream, Cereal | | | | | | | | | | | | | |
| *Below the significance level of 0.05 | | | | | | | | | | | | | |
