# Supplementary material for: Examination of food consumption in United States adults and the prevalence of inflammatory bowel disease using National Health Interview Survey 2015
Source: PLoS One. 2020 Apr 23;15(4):e0232157. doi: 10.1371/journal.pone.0232157 (PMC7179926; doi:10.1371/journal.pone.0232157)
Supplement: S5 Table — (DOCX) [file pone.0232157.s005.docx]

| **Supplemental Table 5 Association (OR^f^) of food consumption (full model) and IBD in estimated US population, NHIS 2015^a,b^** | | | | | | | | | | | | |
| --- | --- | --- | --- | --- | --- | --- | --- | --- | --- | --- | --- | --- |
|  | Weighted, Unadjusted | | | Weighted, Adjusted for Demography | | | Weighted, Adjusted for Lifestyle | | | Weighted, Adjusted for Demography and Lifestyle | | |
| Food items^c^ | OR | p-value | 95% CI | OR | p-value | 95% CI | OR | p-value | 95% CI | OR | p-value | 95% CI |
| Milk (cow milk, any type) | 0.67 | 0.020* | (0.4844 - 0.9403) | 0.69 | 0.034* | (0.4928 - 0.9718) | 0.69 | 0.030* | (0.4872 - 0.9638) | 0.70 | 0.049* | (0.4969 - 0.9978) |
| Salad (green leafy, lettuce) | 0.76 | 0.170 | (0.5132 - 1.1257) | 0.73 | 0.112 | (0.4885 - 1.0776) | 0.76 | 0.168 | (0.5090 - 1.1256) | 0.72 | 0.1080 | (0.4828 - 1.0756) |
| Popcorn | 0.73 | 0.029* | (0.5445 - 0.9674) | 0.72 | 0.025* | (0.5407 - 0.9599) | 0.73 | 0.034* | (0.5494 - 0.9765) | 0.73 | 0.031* | (0.5477 - 0.9710) |
| Vegetables^d^ | 0.80 | 0.412 | (0.4740 - 1.3591) | 0.74 | 0.271 | (0.4372 - 1.2628) | 0.77 | 0.323 | (0.4532 - 1.2987) | 0.72 | 0.2200 | (0.4214 - 1.2208) |
| Whole grain bread | 0.94 | 0.693 | (0.7002 - 1.2677) | 0.97 | 0.843 | (0.7192 - 1.3088) | 0.95 | 0.756 | (0.6993 - 1.2968) | 0.97 | 0.8730 | (0.7139 - 1.3314) |
| Fruit juices (100% pure fruit juice) | 0.83 | 0.211 | (0.6121 - 1.1151) | 0.88 | 0.408 | (0.6493 - 1.1923) | 0.83 | 0.225 | (0.6047 - 1.1262) | 0.88 | 0.4140 | (0.6412 - 1.2014) |
| Cookie (i.e. cake, pies, brownies) | 1.18 | 0.402 | (0.8034 - 1.7238) | 1.13 | 0.532 | (0.7736 - 1.6421) | 1.13 | 0.518 | (0.7762 - 1.6509) | 1.08 | 0.6720 | (0.7468 - 1.5721) |
| Salsa (made with tomatoes) | 0.99 | 0.939 | (0.7247 - 1.3473) | 1.17 | 0.384 | (0.8248 - 1.6472) | 1.01 | 0.932 | (0.7429 - 1.3830) | 1.19 | 0.3280 | (0.8404 - 1.6797) |
| Beans | 0.96 | 0.818 | (0.7015 - 1.3232) | 0.90 | 0.530 | (0.6579 - 1.2410) | 0.97 | 0.852 | (0.6954 - 1.3504) | 0.90 | 0.5360 | (0.6499 - 1.2519) |
| Fruits (fresh, frozen, canned) | 1.00 | 0.999 | (0.6080 - 1.6442) | 0.97 | 0.891 | (0.5856 - 1.5927) | 1.07 | 0.815 | (0.6088 - 1.8775) | 1.03 | 0.9230 | (0.5849 - 1.8069) |
| Cereal (hot or cold) | 1.50 | 0.035* | (1.0286 - 2.1811) | 1.40 | 0.084 | (0.9565 - 2.0378) | 1.48 | 0.042* | (1.0145 - 2.1491) | 1.38 | 0.0970 | (0.9437 - 2.0141) |
| Potato (non-fried) | 1.20 | 0.363 | (0.8086 - 1.7843) | 1.03 | 0.877 | (0.6900 - 1.5444) | 1.28 | 0.217 | (0.8636 - 1.9036) | 1.10 | 0.6460 | (0.7356 - 1.6398) |
| Candy (i.e. chocolates) | 1.04 | 0.826 | (0.7112 - 1.5318) | 1.00 | 0.983 | (0.6811 - 1.4808) | 1.07 | 0.719 | (0.7286 - 1.5813) | 1.03 | 0.8700 | (0.6982 - 1.5289) |
| Fries | 1.48 | 0.026* | (1.0468 - 2.0810) | 1.65 | 0.004* | (1.1761 - 2.3275) | 1.42 | 0.044* | (1.0100 - 1.9943) | 1.60 | 0.006* | (1.1421 - 2.2454) |
| Ice cream (frozen desserts) | 0.98 | 0.913 | (0.7080 - 1.3621) | 0.97 | 0.860 | (0.7009 - 1.3459) | 0.98 | 0.893 | (0.7017 - 1.3619) | 0.97 | 0.8360 | (0.6944 - 1.3434) |
| Pizza (frozen, fast food, homemade) | 0.83 | 0.399 | (0.5444 - 1.2750) | 0.95 | 0.806 | (0.6191 - 1.4519) | 0.89 | 0.603 | (0.5816 - 1.3703) | 1.02 | 0.9100 | (0.6664 - 1.5766) |
| Regular soda or pop^e^ | 0.93 | 0.678 | (0.6726 - 1.2948) | 1.05 | 0.781 | (0.7470 - 1.4735) | 0.93 | 0.675 | (0.6594 - 1.3101) | 1.05 | 0.7830 | (0.7372 - 1.4985) |
| Red meat | 0.98 | 0.931 | (0.6079 - 1.5770) | 0.96 | 0.881 | (0.5997 - 1.5514) | 1.00 | 0.996 | (0.6157 - 1.6286) | 0.99 | 0.9540 | (0.6081 - 1.5982) |
| Brown rice | 0.92 | 0.567 | (0.7013 - 1.2149) | 1.01 | 0.962 | (0.7650 - 1.3247) | 0.92 | 0.534 | (0.6919 - 1.2108) | 1.00 | 0.9870 | (0.7534 - 1.3212) |
| Cheese (excludes cheese on pizza) | 1.21 | 0.448 | (0.7401 - 1.9736) | 1.13 | 0.620 | (0.6912 - 1.8558) | 1.19 | 0.488 | (0.7279 - 1.9411) | 1.11 | 0.6740 | (0.6786 - 1.8208) |
| Sports and energy drinks^e^ | 1.06 | 0.675 | (0.8012 - 1.4076) | 1.43 | 0.021* | (1.0549 - 1.9355) | 1.08 | 0.612 | (0.8076 - 1.4363) | 1.46 | 0.016* | (1.0730 - 1.9746) |
| Tomato sauce | 0.87 | 0.484 | (0.5797 - 1.2954) | 0.84 | 0.402 | (0.5586 - 1.2638) | 0.85 | 0.439 | (0.5672 - 1.2795) | 0.82 | 0.3600 | (0.5453 - 1.2473) |
| Donut (i.e. Danish, pastries, muffins) | 1.04 | 0.804 | (0.7739 - 1.3918) | 1.03 | 0.855 | (0.7658 - 1.3790) | 0.98 | 0.915 | (0.7253 - 1.3341) | 0.98 | 0.8830 | (0.7207 - 1.3259) |
| Processed meat | 1.20 | 0.321 | (0.8392 - 1.7055) | 1.14 | 0.477 | (0.7914 - 1.6478) | 1.26 | 0.220 | (0.8718 - 1.8116) | 1.19 | 0.3490 | (0.8228 - 1.7350) |
| Fruit drinks (sweetened with sugar)^e^ | 1.04 | 0.780 | (0.7836 - 1.3836) | 1.12 | 0.432 | (0.8467 - 1.4738) | 1.02 | 0.900 | (0.7624 - 1.3613) | 1.10 | 0.5100 | (0.8279 - 1.4616) |
| Coffee or tea (sugar or honey added)^e^ | 0.96 | 0.738 | (0.7283 - 1.2523) | 1.00 | 0.985 | (0.7598 - 1.3092) | 0.96 | 0.787 | (0.7346 - 1.2636) | 1.00 | 0.9890 | (0.7641 - 1.3139) |
| Age |  |  |  | 1.24 | 0.000 | (1.1357 - 1.3511) |  |  |  | 1.25 | 0.0000 | (1.1460 - 1.3606) |
| Race |  |  |  | 0.80 | 0.076 | (0.6322 - 1.0234) |  |  |  | 0.80 | 0.0800 | (0.6285 - 1.0267) |
| Sex |  |  |  | 1.41 | 0.017 | (1.0634 - 1.8794) |  |  |  | 1.41 | 0.0180 | (1.0610 - 1.8718) |
| Ethnicity |  |  |  | 0.85 | 0.422 | (0.5746 - 1.2620) |  |  |  | 0.89 | 0.5510 | (0.6072 - 1.3056) |
| Poverty status |  |  |  | 0.82 | 0.047 | (0.6785 - 0.9977) |  |  |  | 0.83 | 0.0640 | (0.6849 - 1.0107) |
| Region |  |  |  | 0.94 | 0.387 | (0.8137 - 1.0834) |  |  |  | 0.94 | 0.4180 | (0.8190 - 1.0866) |
| Alcohol user status |  |  |  |  |  |  | 0.90 | 0.476 | (0.6798 - 1.1980) | 0.91 | 0.5120 | (0.6905 - 1.2034) |
| Alcohol consumption status |  |  |  |  |  |  | 0.99 | 0.955 | (0.8048 - 1.2276) | 1.01 | 0.9260 | (0.8241 - 1.2370) |
| Smoking |  |  |  |  |  |  | 1.07 | 0.159 | (0.9752 - 1.1643) | 1.03 | 0.4970 | (0.9383 - 1.1400) |
| BMI |  |  |  |  |  |  | 0.94 | 0.462 | (0.8071 - 1.1025) | 0.91 | 0.2590 | (0.7831 - 1.0682) |
|  |  |  |  |  |  |  |  |  |  |  |  |  |
|  |  |  |  |  |  |  |  |  |  |  |  |  |
| ^a^Weighted using sample weight [wtfa_sa]. Logistic regression with IBD as outcome; Data source: Sample Adult Cancer file from 2015 NHIS Data release source (https://www.cdc.gov/nchs/nhis/nhis_2015_data_release.htm) | | | | | | | | | | | | |
| ^b^Additional details in survey questions can be found in NHIS 2015 Data release website: ftp://ftp.cdc.gov/pub/Health_Statistics/NCHS/Dataset_Documentation/NHIS/2015/cancerxx_layout.pdf | | | | | | | | | | | | |
| ^c^Order of predictors in full model based on Pearson Chi-square test | | | | | | | | | | | | |
| ^d^Vegetables other than lettuce salads, potatoes, cooked beans in which participant already answered to in previous questions. | | | | | | | | | | | | |
| ^e^Diet items excludes artificially sweetened or sugar-free kinds | | | | | | | | | | | | |
| ^f^Odds Ratio: (Odds of having IBD in those consuming food item/Odds of having IBD in those never consumed the food item) | | | | | | | | | | | | |
| *Statistically significant; Below the significance level of 0.05 | | | | | | | | | | | | |
|  | | | | | | | | | | | | |
|  | | | | | | | | | | | | |
|  | | | | | | | | | | | | |
|  | | | | | | | | | | | | |
|  | | | | | | | | | | | | |
|  | | | | | | | | | | | | |
