# Supplementary material for: Examination of food consumption in United States adults and the prevalence of inflammatory bowel disease using National Health Interview Survey 2015
Source: PLoS One. 2020 Apr 23;15(4):e0232157. doi: 10.1371/journal.pone.0232157 (PMC7179926; doi:10.1371/journal.pone.0232157)
Supplement: S6 Table — (DOCX) [file pone.0232157.s006.docx]

| **Supplemental Table 6 Association (OR^f^) of food consumption frequency (full model) and IBD in estimated US population, NHIS 2015^a,b^** | | | | | | | | | | | | |
| --- | --- | --- | --- | --- | --- | --- | --- | --- | --- | --- | --- | --- |
|  | Weighted, unadjusted | | | Weighted, Adjusted for Demography | | | Weighted, Adjusted for Lifestyle | | | Weighted, Adjusted for  Demography and Lifestyle | | |
| Food items^c^ | OR | p-value | 95% CI | OR | p-value | 95% CI | OR | p-value | 95% CI | OR | p-value | 95% CI |
| Popcorn | 0.97 | 0.085 | (0.9472 - 1.0035) | 0.97 | 0.061 | (0.9464 - 1.0012) | 0.98 | 0.099 | (0.9488 - 1.0045) | 0.97 | 0.075 | (0.9480 - 1.0026) |
| Cereal (hot or cold) | 1.00 | 0.298 | (0.9964 - 1.0117) | 1.00 | 0.400 | (0.9957 - 1.0109) | 1.00 | 0.305 | (0.9964 - 1.0116) | 1.00 | 0.420 | (0.9955 - 1.0108) |
| Brown rice | 0.99 | 0.563 | (0.9776 - 1.0124) | 1.00 | 0.770 | (0.9871 - 1.0176) | 0.99 | 0.534 | (0.9771 - 1.0121) | 1.00 | 0.818 | (0.9866 - 1.0173) |
| Whole grain bread | 1.00 | 0.652 | (0.9913 - 1.0055) | 1.00 | 0.500 | (0.9904 - 1.0047) | 1.00 | 0.595 | (0.9910 - 1.0052) | 1.00 | 0.445 | (0.9900 - 1.0044) |
| Salad (green leafy, lettuce) | 1.00 | 0.834 | (0.9882 - 1.0097) | 1.00 | 0.803 | (0.9877 - 1.0097) | 1.00 | 0.929 | (0.9888 - 1.0103) | 1.00 | 0.875 | (0.9881 - 1.0103) |
| Salsa (made with tomatoes) | 0.99 | 0.228 | (0.9757 - 1.0059) | 1.00 | 0.606 | (0.9812 - 1.0111) | 0.99 | 0.248 | (0.9762 - 1.0063) | 1.00 | 0.578 | (0.9810 - 1.0107) |
| Pizza (frozen, fast food, homemade) | 0.97 | 0.167 | (0.9210 - 1.0144) | 0.98 | 0.423 | (0.9441 - 1.0245) | 0.97 | 0.204 | (0.9276 - 1.0162) | 0.99 | 0.568 | (0.9517 - 1.0276) |
| Fruit juices (100% pure fruit juice) | 1.00 | 0.383 | (0.9876 - 1.0048) | 1.00 | 0.415 | (0.9878 - 1.0051) | 1.00 | 0.404 | (0.9875 - 1.0051) | 1.00 | 0.432 | (0.9876 - 1.0054) |
| Potato (non-fried) | 1.01 | 0.124 | (0.9973 - 1.0226) | 1.00 | 0.531 | (0.9909 - 1.0178) | 1.01 | 0.090 | (0.9984 - 1.0231) | 1.01 | 0.439 | (0.9921 - 1.0184) |
| Tomato sauce | 1.01 | 0.285 | (0.9923 - 1.0266) | 1.01 | 0.290 | (0.9924 - 1.0258) | 1.01 | 0.325 | (0.9915 - 1.0260) | 1.01 | 0.344 | (0.9914 - 1.0251) |
| Beans | 1.00 | 0.443 | (0.9824 - 1.0078) | 0.99 | 0.464 | (0.9815 - 1.0086) | 1.00 | 0.492 | (0.9824 - 1.0086) | 1.00 | 0.507 | (0.9815 - 1.0093) |
| Fries | 1.00 | 0.427 | (0.9936 - 1.0153) | 1.01 | 0.071 | (0.9992 - 1.0182) | 1.00 | 0.544 | (0.9924 - 1.0145) | 1.01 | 0.088 | (0.9988 - 1.0176) |
| Fruits (fresh, frozen, canned) | 1.00 | 0.834 | (0.9935 - 1.0053) | 1.00 | 0.785 | (0.9933 - 1.0051) | 1.00 | 0.808 | (0.9931 - 1.0054) | 1.00 | 0.741 | (0.9927 - 1.0052) |
| Vegetables^d^ | 0.99 | 0.102 | (0.9867 - 1.0012) | 0.99 | 0.083 | (0.9858 - 1.0009) | 0.99 | 0.122 | (0.9871 - 1.0015) | 0.99 | 0.106 | (0.9863 - 1.0013) |
| Milk (cow milk, any type) | 1.00 | 0.323 | (0.9855 - 1.0048) | 1.00 | 0.310 | (0.9856 - 1.0046) | 1.00 | 0.324 | (0.9853 - 1.0049) | 1.00 | 0.314 | (0.9855 - 1.0047) |
| Cheese (excludes cheese on pizza) | 1.01 | 0.001* | (1.0035 - 1.0125) | 1.01 | <0.001* | (1.0040 - 1.0130) | 1.01 | <0.001* | (1.0040 - 1.0129) | 1.01 | <0.001* | (1.0046 - 1.0133) |
| Ice cream (frozen desserts) | 1.01 | 0.103 | (0.9980 - 1.0215) | 1.01 | 0.212 | (0.9955 - 1.0204) | 1.01 | 0.127 | (0.9974 - 1.0212) | 1.01 | 0.239 | (0.9950 - 1.0202) |
| Red meat | 0.99 | 0.316 | (0.9825 - 1.0057) | 1.00 | 0.433 | (0.9844 - 1.0068) | 0.99 | 0.342 | (0.9829 - 1.0060) | 1.00 | 0.464 | (0.9849 - 1.0070) |
| Processed meat | 1.00 | 0.513 | (0.9847 - 1.0078) | 1.00 | 0.579 | (0.9855 - 1.0082) | 1.00 | 0.681 | (0.9861 - 1.0092) | 1.00 | 0.734 | (0.9869 - 1.0093) |
| Cookies (i.e. cake, pies, brownies) | 1.00 | 0.661 | (0.9908 - 1.0146) | 1.00 | 0.921 | (0.9882 - 1.0132) | 1.00 | 0.686 | (0.9905 - 1.0146) | 1.00 | 0.922 | (0.9879 - 1.0135) |
| Candy (i.e. chocolates) | 1.00 | 0.375 | (0.9957 - 1.0116) | 1.00 | 0.478 | (0.9948 - 1.0112) | 1.00 | 0.323 | (0.9961 - 1.0118) | 1.00 | 0.461 | (0.9948 - 1.0115) |
| Coffee or tea (sugar or honey added) | 1.00 | 0.357 | (0.9978 - 1.0061) | 1.00 | 0.410 | (0.9977 - 1.0056) | 1.00 | 0.383 | (0.9976 - 1.0062) | 1.00 | 0.452 | (0.9975 - 1.0056) |
| Donut (i.e. Danish, pastries, muffins) | 1.00 | 0.772 | (0.9861 - 1.0190) | 1.00 | 0.883 | (0.9847 - 1.0181) | 1.00 | 0.838 | (0.9850 - 1.0188) | 1.00 | 0.964 | (0.9834 - 1.0177) |
| Sports and energy drinks | 0.99 | 0.417 | (0.9826 - 1.0073) | 1.00 | 0.830 | (0.9914 - 1.0108) | 1.00 | 0.455 | (0.9831 - 1.0077) | 1.00 | 0.797 | (0.9917 - 1.0109) |
| Regular soda or pop | 1.01 | 0.007* | (1.0016 - 1.0101) | 1.01 | 0.003* | (1.0022 - 1.0103) | 1.01 | 0.012* | (1.0012 - 1.0101) | 1.01 | 0.004* | (1.0020 - 1.0104) |
| Fruit drinks (sweetened with sugar) | 1.00 | 0.621 | (0.9861 - 1.0084) | 1.00 | 0.836 | (0.9890 - 1.0090) | 1.00 | 0.571 | (0.9855 - 1.0081) | 1.00 | 0.805 | (0.9885 - 1.0090) |
| Age |  |  |  | 1.20 | 0.000 | (1.1120 - 1.2956) |  |  |  | 1.21 | 0.000 | (1.1227 - 1.3054) |
| Race |  |  |  | 0.81 | 0.089 | (0.6379 - 1.0326) |  |  |  | 0.81 | 0.095 | (0.6335 - 1.0372) |
| Sex |  |  |  | 1.30 | 0.066 | (0.9825 - 1.7109) |  |  |  | 1.30 | 0.062 | (0.9872 - 1.7206) |
| Ethnicity |  |  |  | 0.87 | 0.538 | (0.5608 - 1.3533) |  |  |  | 0.91 | 0.685 | (0.5951 - 1.4068) |
| Poverty status |  |  |  | 0.85 | 0.079 | (0.7048 - 1.0195) |  |  |  | 0.86 | 0.104 | (0.7092 - 1.0328) |
| Region |  |  |  | 0.97 | 0.636 | (0.8341 - 1.1175) |  |  |  | 0.97 | 0.706 | (0.8420 - 1.1236) |
| Alcohol user status |  |  |  |  |  |  | 0.90 | 0.482 | (0.6807 - 1.1994) | 0.93 | 0.585 | (0.7039 - 1.2195) |
| Alcohol consumption status |  |  |  |  |  |  | 1.00 | 0.963 | (0.8156 - 1.2380) | 1.02 | 0.860 | (0.8350 - 1.2408) |
| Smoking |  |  |  |  |  |  | 1.06 | 0.244 | (0.9630 - 1.1591) | 1.03 | 0.565 | (0.9300 - 1.1418) |
| BMI |  |  |  |  |  |  | 0.95 | 0.499 | (0.8064 - 1.1108) | 0.92 | 0.300 | (0.7834 - 1.0783) |
|  |  |  |  |  |  |  |  |  |  |  |  |  |
| ^a^Weighted using sample weight [wtfa_sa]. Logistic regression with IBD as outcome; Data source: Sample Adult Cancer file from 2015 NHIS Data release source (https://www.cdc.gov/nchs/nhis/nhis_2015_data_release.htm) | | | | | | | | | | | | |
| ^b^Additional details in survey questions can be found in NHIS 2015 Data release website: ftp://ftp.cdc.gov/pub/Health_Statistics/NCHS/Dataset_Documentation/NHIS/2015/cancerxx_layout.pdf | | | | | | | | | | | | |
| ^c^The order of each food item in full model are based on its association with IBD found by Wilcoxon-Mann-Whitney test. | | | | | | | | | | | | |
| ^d^Vegetables other than lettuce salads, potatoes, cooked beans in which participant already answered to in previous questions. | | | | | | | | | | | | |
| ^e^Food items in this group exclude artificially sweetened or sugar-free kinds | | | | | | | | | | | | |
| ^f^Odds of having IBD with every unit increase in consumption of respective food item | | | | | | | | | | | | |
| *Statistically significant; Below the significance level of 0.05 | | | | | | | | | | | | |
