# Supplementary material for: Examination of food consumption in United States adults and the prevalence of inflammatory bowel disease using National Health Interview Survey 2015
Source: PLoS One. 2020 Apr 23;15(4):e0232157. doi: 10.1371/journal.pone.0232157 (PMC7179926; doi:10.1371/journal.pone.0232157)
Supplement: S7 Table — (DOCX) [file pone.0232157.s007.docx]

| **Supplemental Table 7 Population size and average (Median) monthly food intake frequency for US sample adults and estimated adult population, NHIS 2015^a,b^** | | | | | | | |
| --- | --- | --- | --- | --- | --- | --- | --- |
|  |  | Sample Adult Population | | | General Population | | |
|  |  | Unweighted, Unadjusted | | | Weighted, Unadjusted | | |
| Food groups^c^ | Food items | N | Median | IQR | N | Median | IQR |
| Whole wheat grains | Popcorn | 31,649 | 1.000 | (0.000 - 2.000) | 226,750,764 | 1.000 | (0.000 - 2.000) |
|  | Cereal (hot or cold)^f^ | 31,831 | 5.000 | (0.000 - 17.333) | 228,078,386 | 5.000 | (0.000 - 17.333) |
|  | Brown rice | 31,711 | 0.000 | (0.000 - 4.333) | 227,158,212 | 1.000 | (0.000 - 4.333) |
|  | Whole grain bread | 31,640 | 8.666 | (1.000 - 30.000) | 226,741,285 | 8.666 | (1.000 - 25.000) |
| Fruits and vegetables | Fries | 31,737 | 4.000 | (1.000 - 8.667) | 227,462,700 | 4.333 | (1.000 - 8.667) |
|  | Salad (green leafy, lettuce) | 31,770 | 13.000 | (4.333 - 21.666) | 227,675,083 | 13.000 | (4.333 - 21.666) |
|  | Fruit juices (100% pure fruit juice) | 31,765 | 4.333 | (0.000 - 15.000) | 227,626,105 | 4.333 | (0.000 - 15.000) |
|  | Vegetables^d^ | 31,687 | 21.667 | (10.000 - 30.333) | 227,072,944 | 21.667 | (10.000 - 30.333) |
|  | Potato (non-fried) | 31,700 | 4.333 | (2.000 - 8.666) | 227,185,918 | 4.333 | (2.000 - 8.666) |
|  | Fruits (fresh, frozen, canned) | 31,768 | 21.666 | (8.667 - 30.333) | 227,663,492 | 21.666 | (8.667 - 30.333) |
|  | Pizza (frozen, fast food, homemade)^f^ | 31,723 | 2.000 | (1.000 - 4.333) | 227,304,506 | 2.000 | (1.000 - 4.333) |
|  | Tomato sauce | 31,661 | 3.000 | (1.000 - 4.333) | 226,790,518 | 3.000 | (1.000 - 4.333) |
|  | Salsa (made with tomatoes) | 31,695 | 2.000 | (0.000 - 5.000) | 227,025,710 | 2.000 | (0.000 - 5.000) |
|  | Beans | 31,702 | 4.000 | (1.000 - 8.666) | 227,049,415 | 4.000 | (1.000 - 8.666) |
| Dairy | Milk (cow milk, any type) | 31,804 | 13.000 | (1.000 - 30.333) | 227,906,932 | 13.000 | (1.000 - 30.333) |
|  | Cheese (excludes cheese on pizza) | 31,668 | 13.000 | (4.333 - 30.000) | 226,905,312 | 13.000 | (5.000 - 30.000) |
|  | Pizza (frozen, fast food, homemade)^f^ | 31,723 | 2.000 | (1.000 - 4.333) | 227,304,506 | 2.000 | (1.000 - 4.333) |
|  | Ice cream (frozen desserts)^f^ | 31,646 | 2.000 | (0.000 - 5.000) | 226,704,695 | 2.000 | (0.000 - 5.000) |
| Meat | Processed meat | 31,668 | 4.333 | (1.000 - 8.666) | 226,915,214 | 4.333 | (1.000 - 8.666) |
|  | Red meat | 31,670 | 8.666 | (4.333 - 17.333) | 226,911,625 | 8.666 | (4.333 - 17.333) |
| Sweetened food/drinks^e^ | Cereal (hot or cold)^f^ | 31,831 | 5.000 | (0.000 - 17.333) | 228,078,386 | 5.000 | (0.000 - 17.333) |
|  | Cookies (i.e. cake, pies, brownies) | 31,635 | 3.000 | (0.000 - 8.666) | 226,659,263 | 3.000 | (0.000 - 8.666) |
|  | Donut (i.e. Danish, pastries, muffins) | 31,658 | 1.000 | (0.000 - 4.333) | 226,794,796 | 1.000 | (0.000 - 4.333) |
|  | Candy (i.e. chocolates) | 31,646 | 4.333 | (1.000 - 13.000) | 226,690,983 | 4.333 | (1.000 - 13.000) |
|  | Sports and energy drinks | 31,805 | 0.000 | (0.000 - 1.000) | 227,921,423 | 0.000 | (0.000 - 1.000) |
|  | Coffee or tea (sugar or honey added) | 31,792 | 4.000 | (0.000 - 30.333) | 227,814,182 | 4.333 | (0.000 - 30.333) |
|  | Fruit drinks (sweetened with sugar) | 31,785 | 0.000 | (0.000 - 1.000) | 227,744,976 | 0.000 | (0.000 - 1.000) |
|  | Regular soda or pop | 31,809 | 1.000 | (0.000 - 13.000) | 227,963,515 | 2.000 | (0.000 - 13.000) |
|  | Ice cream (frozen desserts)^f^ | 31,646 | 2.000 | (0.000 - 5.000) | 226,704,695 | 2.000 | (0.000 - 5.000) |
|  |  |  |  |  |  |  |  |
|  |  |  |  |  |  |  |  |
|  |  |  |  |  |  |  |  |
|  |  |  |  |  |  |  |  |
| ^a^Weighted using sample weight [wtfa_sa]; Data source: Sample Adult Cancer file from 2015 NHIS Data release source (https://www.cdc.gov/nchs/nhis/nhis_2015_data_release.htm) | | | | | | | |
| ^b^Additional details in survey questions can be found in NHIS 2015 Data release website: ftp://ftp.cdc.gov/pub/Health_Statistics/NCHS/Dataset_Documentation/NHIS/2015/cancerxx_layout.pdf | | | | | | | |
| ^c^Food groups are based on the relationship previously established according the dietary guidelines. Details can be found on https://epi.grants.cancer.gov/nhanes/dietscreen/relationship.html. | | | | | | | |
| ^d^Vegetables other than lettuce salads, potatoes, cooked beans in which participant already answered to in previous questions. | | | | | | | |
| ^e^Food items in this group excludes artificially sweetened or sugar-free kinds | | | | | | | |
| ^f^Food items appear in more than one food groups: Pizza, Ice cream, Cereal | | | | | | | |
| IQR (Interquartile Range) | | | | | | | |
