# Supplementary material for: Examination of food consumption in United States adults and the prevalence of inflammatory bowel disease using National Health Interview Survey 2015
Source: PLoS One. 2020 Apr 23;15(4):e0232157. doi: 10.1371/journal.pone.0232157 (PMC7179926; doi:10.1371/journal.pone.0232157)
Supplement: S8 Table — (DOCX) [file pone.0232157.s008.docx]

| **Supplemental Table 8 Comparison in point prevalence of colitis in estimated subpopulation with different average (Median) monthly food intake rate, NHIS 2015^a,b^** | | | | | | | | |
| --- | --- | --- | --- | --- | --- | --- | --- | --- |
|  |  |  | Weighted, Unadjusted | | | |  | |
|  |  |  | Point prevalence of IBD | | | |  |  |
|  |  | Monthly median^b^ | > Median | | <= Median | | Adjusted Wald Test^g^ | |
| Food groups^c^ | Food items |  | % | 95% CI | % | 95% CI | F-test | p-value |
| Whole wheat grains | Popcorn | 1.00 | 1.23 | (1.2265 - 1.2314) | 1.33 | (1.3274 - 1.3310) | 0.28 | 0.599 |
|  | Cereal (hot or cold) | 5.00 | 1.29 | (1.2886 - 1.2928) | 1.27 | (1.2705 - 1.2745) | 0.01 | 0.918 |
|  | Brown rice^f^ | 1.00 | 1.19 | (1.1878 - 1.1921) | 1.37 | (1.3667 - 1.3707) | 1.24 | 0.266 |
|  | Whole grain bread | 8.67 | 1.41 | (1.4101 - 1.4144) | 1.18 | (1.1792 - 1.1831) | 2.13 | 0.146 |
| Fruits and vegetables | Fries | 4.33 | 1.34 | (1.3416 - 1.3458) | 1.22 | (1.2188 - 1.2229) | 0.53 | 0.466 |
|  | Salad (green leafy, lettuce) | 13.00 | 1.24 | (1.2373 - 1.2418) | 1.32 | (1.3199 - 1.3237) | 0.22 | 0.637 |
|  | Fruit juices (100% pure fruit juice) | 4.33 | 1.11 | (1.1075 - 1.1117) | 1.49 | (1.4869 - 1.4914) | 4.52 | 0.034* |
|  | Vegetables^d^ | 21.67 | 1.29 | (1.2881 - 1.2923) | 1.30 | (1.2935 - 1.2976) | 0.00 | 0.976 |
|  | Potato (non-fried) | 4.33 | 1.33 | (1.3264 - 1.3302) | 1.24 | (1.2369 - 1.2414) | 0.26 | 0.611 |
|  | Fruits (fresh, frozen, canned) | 21.67 | 1.24 | (1.2428 - 1.2470) | 1.32 | (1.3229 - 1.3270) | 0.22 | 0.637 |
|  | Pizza (frozen, fast food, homemade) | 2.00 | 1.17 | (1.1696 - 1.1739) | 1.38 | (1.3806 - 1.3846) | 1.58 | 0.210 |
|  | Tomato sauce | 3.00 | 1.33 | (1.3229 - 1.3273) | 1.26 | (1.2625 - 1.2665) | 0.15 | 0.700 |
|  | Salsa (made with tomatoes) | 2.00 | 1.32 | (1.3187 - 1.3232) | 1.27 | (1.2690 - 1.2729) | 0.09 | 0.765 |
|  | Beans | 4.00 | 1.23 | (1.2302 - 1.2343) | 1.35 | (1.3510 - 1.3552) | 0.53 | 0.466 |
| Dairy | Milk (cow milk, any type) | 13.00 | 1.15 | (1.1524 - 1.1566) | 1.39 | (1.3894 - 1.3935) | 2.07 | 0.151 |
|  | Cheese (excludes cheese on pizza) | 13.00 | 1.56 | (1.5557 - 1.5606) | 1.08 | (1.0815 - 1.0851) | 8.36 | 0.004* |
|  | Pizza (frozen, fast food, homemade) | 2.00 | 1.17 | (1.1696 - 1.1739) | 1.38 | (1.3806 - 1.3846) | 1.58 | 0.210 |
|  | Ice cream (frozen desserts) | 2.00 | 1.43 | (1.4317 - 1.4363) | 1.18 | (1.1786 - 1.1824) | 2.23 | 0.137 |
| Meat | Processed meat | 4.33 | 1.32 | (1.3143 - 1.3184) | 1.26 | (1.2628 - 1.2670) | 0.10 | 0.750 |
|  | Red meat | 8.67 | 1.26 | (1.2538 - 1.2581) | 1.32 | (1.3214 - 1.3254) | 0.18 | 0.675 |
| Sweetened food/drinks^e^ | Cereal (hot or cold) | 5.00 | 1.29 | (1.2886 - 1.2928) | 1.27 | (1.2705 - 1.2745) | 0.01 | 0.918 |
|  | Cookies (i.e. cake, pies, brownies) | 3.00 | 1.48 | (1.4794 - 1.4840) | 1.13 | (1.1311 - 1.1348) | 4.13 | 0.043* |
|  | Donut (i.e. Danish, pastries, muffins) | 1.00 | 1.25 | (1.2466 - 1.2511) | 1.33 | (1.3265 - 1.3304) | 0.24 | 0.622 |
|  | Candy (i.e. chocolates) | 4.33 | 1.36 | (1.3608 - 1.3648) | 1.21 | (1.1207 - 1.2109) | 0.85 | 0.356 |
|  | Sports and energy drinks^f^ | 0.00 | 1.34 | (1.3401 - 1.3457) | 1.27 | (1.2647 - 1.2682) | 0.20 | 0.656 |
|  | Coffee or tea (sugar or honey added) | 4.33 | 1.23 | (1.2236 - 1.2277) | 1.35 | (1.3501 - 1.3543) | 0.62 | 0.431 |
|  | Fruit drinks (sweetened with sugar)^f^ | 0.00 | 1.35 | (1.3492 - 1.3549) | 1.26 | (1.2629 - 1.2664) | 0.28 | 0.596 |
|  | Regular soda or pop | 2.00 | 1.31 | (1.3076 - 1.3118) | 1.27 | (1.2698 - 1.2739) | 0.05 | 0.824 |
|  | Ice cream (frozen desserts) | 2.00 | 1.43 | (1.4317 - 1.4363) | 1.18 | (1.1786 - 1.1824) | 2.23 | 0.137 |
|  | Regular soda or pop | 2.00 | 1.28 | (1.2764 - 1.2807) | 1.30 | (1.2994 - 1.3034) | 0.02 | 0.890 |
|  | | | | | | | | |
|  | | | | | | | | |
| ^a^Weighted using sample weight [wtfa_sa]; Data source: Sample Adult Cancer file from 2015 NHIS Data release source (https://www.cdc.gov/nchs/nhis/nhis_2015_data_release.htm) | | | | | | | | |
| ^b^Additional details in survey questions can be found in NHIS 2015 Data release website: ftp://ftp.cdc.gov/pub/Health_Statistics/NCHS/Dataset_Documentation/NHIS/2015/cancerxx_layout.pdf | | | | | | | | |
| ^c^Food groups are based on the relationship previously established according the dietary guidelines. Details can be found on https://epi.grants.cancer.gov/nhanes/dietscreen/relationship.html. | | | | | | | | |
| ^d^Vegetables other than lettuce salads, potatoes, cooked beans in which participant already answered to in previous questions. | | | | | | | | |
| ^e^Food items in this group excludes artificially sweetened or sugar-free kinds | | | | | | | | |
| ^f^The median for these diet items are 0, or none. Equivalent to having never consumed in past month. | | | | | | | | |
| ^g^Point prevalence estimations between IBD population eating > Median was compared to IBD population eating ≤Median | | | | | | | | |
| *Statistically significant; Below the significance level of 0.05 | | | | | | | | |
