# Supplementary material for: Examination of food consumption in United States adults and the prevalence of inflammatory bowel disease using National Health Interview Survey 2015
Source: PLoS One. 2020 Apr 23;15(4):e0232157. doi: 10.1371/journal.pone.0232157 (PMC7179926; doi:10.1371/journal.pone.0232157)
Supplement: S9 Table — (DOCX) [file pone.0232157.s009.docx]

| **Supplemental Table 9 Comparison in association (OR^h^) of IBD and different average food intake in estimated US population, NHIS 2015^a,b^** | | | | | | | | | | | | | |
| --- | --- | --- | --- | --- | --- | --- | --- | --- | --- | --- | --- | --- | --- |
|  |  | Weighted, Unadjusted | | | Weighted, Adjusted for Demography^f^ | | | Weighted, Adjusted for Lifestyle^g^ | | | Weighted, Adjusted for  Demography and Lifestyle^f,g^ | | |
|  |  | <=Median | | | <=Median | | | <=Median | | | <=Median | | |
| Food groups^c^ | Food items | OR | P-value | 95% CI | OR | P-value | 95% CI | OR | P-value | 95% CI | OR | P-value | 95% CI |
| Whole wheat grains | Popcorn | 1.08 | 0.603 | (0.8015 - 1.4624) | 1.09 | 0.581 | (0.8059 - 1.4685) | 1.08 | 0.615 | (0.7947 - 1.4742) | 1.09 | 0.597 | (0.7983 - 1.4783) |
|  | Cereal (hot or cold)^i^ | 0.99 | 0.917 | (0.7504 - 1.2949) | 1.05 | 0.730 | (0.7980 - 1.3794) | 1.00 | 0.977 | (0.7582 - 1.3301) | 1.07 | 0.641 | (0.8066 - 1.4173) |
|  | Brown rice | 1.14 | 0.337 | (0.8731 - 1.4939) | 1.06 | 0.688 | (0.8059 - 1.3862) | 1.15 | 0.318 | (0.8767 - 1.4965) | 1.07 | 0.624 | (0.8140 - 1.4088) |
|  | Whole grain bread | 0.83 | 0.148 | (0.6528 - 1.0667) | 0.86 | 0.224 | (0.6697 - 1.0987) | 0.81 | 0.104 | (0.6284 - 1.0446) | 0.84 | 0.170 | (0.6468 - 1.0801) |
| Fruits and vegetables | Fries | 0.91 | 0.462 | (0.7003 - 1.1759) | 0.79 | 0.091 | (0.6083 - 1.0374) | 0.92 | 0.538 | (0.7097 - 1.1964) | 0.80 | 0.108 | (0.6123 - 1.0499) |
|  | Salad (green leafy, lettuce) | 1.07 | 0.640 | (0.8117 - 1.4033) | 1.09 | 0.558 | (0.8192 - 1.4462) | 1.04 | 0.763 | (0.7918 - 1.3743) | 1.08 | 0.618 | (0.8079 - 1.4309) |
|  | Fruit juices (100% pure fruit juice) | 1.35 | 0.031* | (1.0281 - 1.7648) | 1.27 | 0.087 | (0.9660 - 1.6714) | 1.36 | 0.027* | (1.0358 - 1.7846) | 1.28 | 0.076 | (0.9742 - 1.6910) |
|  | Vegetables^d^ | 1.00 | 0.976 | (0.7623 - 1.3229) | 1.06 | 0.678 | (0.7944 - 1.4240) | 0.99 | 0.969 | (0.7552 - 1.3096) | 1.05 | 0.719 | (0.7876 - 1.4127) |
|  | Potato (non-fried) | 0.93 | 0.613 | (0.7089 - 1.2253) | 1.00 | 0.990 | (0.7632 - 1.3060) | 0.91 | 0.491 | (0.6895 - 1.1956) | 0.97 | 0.831 | (0.7416 - 1.2717) |
|  | Pizza (frozen, fast food, homemade)^i^ | 1.18 | 0.217 | (0.9058 - 1.5437) | 1.03 | 0.832 | (0.7909 - 1.3382) | 1.14 | 0.352 | (0.8675 - 1.4886) | 0.98 | 0.856 | (0.7477 - 1.2735) |
|  | Fruits (fresh, frozen, canned) | 1.07 | 0.639 | (0.8179 - 1.3870) | 1.10 | 0.469 | (0.8439 - 1.4439) | 1.07 | 0.595 | (0.8233 - 1.4032) | 1.12 | 0.420 | (0.8525 - 1.4651) |
|  | Tomato sauce | 0.95 | 0.699 | (0.7491 - 1.2141) | 0.92 | 0.504 | (0.7212 - 1.1748) | 0.94 | 0.641 | (0.7369 - 1.2072) | 0.91 | 0.466 | (0.7113 - 1.1681) |
|  | Salsa (made with tomatoes) | 0.96 | 0.764 | (0.7446 - 1.2419) | 0.80 | 0.127 | (0.5998 - 1.0659) | 0.95 | 0.717 | (0.7344 - 1.2368) | 0.80 | 0.134 | (0.6008 - 1.0707) |
|  | Beans | 1.10 | 0.465 | (0.8520 - 1.4189) | 1.06 | 0.650 | (0.8131 - 1.3929) | 1.08 | 0.575 | (0.8277 - 1.4050) | 1.05 | 0.730 | (0.7954 - 1.3858) |
| Dairy | Milk (cow milk, any type) | 1.21 | 0.154 | (0.9314 - 1.5672) | 1.25 | 0.087 | (0.9670 - 1.6407) | 1.23 | 0.123 | (0.9459 - 1.5880) | 1.27 | 0.074 | (0.9766 - 1.6557) |
|  | Cheese (excludes cheese on pizza) | 0.69 | 0.003* | (0.5418 - 0.8837) | 0.65 | 0.001* | (0.5128 - 0.8366) | 0.66 | 0.001* | (0.5136 - 0.8496) | 0.63 | <0.001* | (0.4906 - 0.8069) |
|  | Pizza (frozen, fast food, homemade)^i^ | 1.18 | 0.217 | (0.9058 - 1.5437) | 1.03 | 0.832 | (0.7909 - 1.3382) | 1.14 | 0.352 | (0.8675 - 1.4886) | 0.98 | 0.856 | (0.7477 - 1.2735) |
|  | Ice cream (frozen desserts)^i^ | 0.82 | 0.131 | (0.6358 - 1.0605) | 0.84 | 0.184 | (0.6555 - 1.0847) | 0.84 | 0.170 | (0.6462 - 1.0804) | 0.86 | 0.226 | (0.6653 - 1.1014) |
| Meat | Processed meat | 0.96 | 0.750 | (0.7484 - 1.2324) | 0.92 | 0.537 | (0.7129 - 1.1934) | 0.93 | 0.553 | (0.7192 - 1.1933) | 0.89 | 0.373 | (0.6862 - 1.1520) |
|  | Red meat | 1.05 | 0.676 | (0.8218 - 1.3529) | 0.99 | 0.950 | (0.7690 - 1.2796) | 1.03 | 0.841 | (0.7962 - 1.3229) | 0.96 | 0.772 | (0.7408 - 1.2497) |
| Sweetened food/drinks^e^ | Cereal (hot or cold)^i^ | 0.99 | 0.917 | (0.7504 - 1.2949) | 1.05 | 0.730 | (0.7980 - 1.3794) | 1.00 | 0.977 | (0.7582 - 1.3301) | 1.07 | 0.641 | (0.8066 - 1.4173) |
|  | Cookies (i.e. cake, pies, brownies) | 0.76 | 0.042* | (0.5860 - 0.9907) | 0.79 | 0.072 | (0.6072 - 1.0220) | 0.79 | 0.079 | (0.6062 - 1.0280) | 0.82 | 0.128 | (0.6278 - 1.0606) |
|  | Donut (i.e. Danish, pastries, muffins) | 1.06 | 0.623 | (0.8288 - 1.3676) | 1.04 | 0.753 | (0.8105 - 1.3367) | 1.11 | 0.439 | (0.8543 - 1.4361) | 1.08 | 0.566 | (0.8322 - 1.3981) |
|  | Coffee or tea (sugar or honey added) | 1.10 | 0.431 | (0.8615 - 1.4165) | 1.05 | 0.720 | (0.8160 - 1.3416) | 0.10 | 0.439 | (0.8601 - 4.4136) | 1.05 | 0.704 | (0.8191 - 1.3433) |
|  | Fruit drinks (sweetened with sugar) | 0.93 | 0.590 | (0.7299 - 1.1966) | 0.82 | 0.121 | (0.6439 - 1.0530) | 0.95 | 0.693 | (0.7376 - 1.2245) | 0.83 | 0.156 | (0.6457 - 1.0732) |
|  | Candy (i.e. chocolates) | 0.89 | 0.358 | (0.6828 - 1.1485) | 0.88 | 0.327 | (0.6801 - 1.1374) | 0.88 | 0.342 | (0.6764 - 1.1458) | 0.88 | 0.324 | (0.6748 - 1.1393) |
|  | Sports and energy drinks | 0.94 | 0.651 | (0.7282 - 1.2194) | 0.68 | 0.010* | (0.5033 - 0.9098) | 0.93 | 0.584 | (0.7104 - 1.2129) | 0.66 | 0.008* | (0.4933 - 0.8961) |
|  | Regular soda or pop | 0.97 | 0.824 | (0.7470 - 1.2614) | 0.84 | 0.238 | (0.6305 - 0.1218) | 0.97 | 0.831 | (0.7380 - 1.2766) | 0.83 | 0.236 | (0.6182 - 1.1261) |
|  | Ice cream (frozen desserts)^i^ | 0.82 | 0.131 | (0.6358 - 1.0605) | 0.84 | 0.184 | (0.6555 - 1.0847) | 0.84 | 0.170 | (0.6462 - 1.0804) | 0.86 | 0.226 | (0.6653 - 1.1014) |
|  |  |  |  |  |  |  |  |  |  |  |  |  |  |
|  |  |  |  |  |  |  |  |  |  |  |  |  |  |
|  |  |  |  |  |  |  |  |  |  |  |  |  |  |
| ^a^Weighted using sample weight [wtfa_sa]. Logistic regression with IBD as outcome; Data source: Sample Adult Cancer file from 2015 NHIS Data release source (https://www.cdc.gov/nchs/nhis/nhis_2015_data_release.htm) | | | | | | | | | | | | | |
| ^b^Additional details in survey questions can be found in NHIS 2015 Data release website: ftp://ftp.cdc.gov/pub/Health_Statistics/NCHS/Dataset_Documentation/NHIS/2015/cancerxx_layout.pdf | | | | | | | | | | | | | |
| ^c^Food groups are based on the relationship previously established according the dietary guidelines. Details can be found on https://epi.grants.cancer.gov/nhanes/dietscreen/relationship.html. | | | | | | | | | | | | | |
| ^d^Vegetables other than lettuce salads, potatoes, cooked beans in which participant already answered to in previous questions. | | | | | | | | | | | | | |
| ^e^Food items in this group excludes artificially sweetened or sugar-free kinds | | | | | | | | | | | | | |
| ^f^Each food item adjusted for demographic factors: Age, race, poverty status, sex, ethnicity, region | | | | | | | | | | | | | |
| ^g^Each food item adjusted for lifestyle factors: Smoking, alcohol user status, alcohol consumption rate, BMI | | | | | | | | | | | | | |
| ^h^Odds Ratio: (Odds of having IBD in those consuming at ≤ Median monthly rate /Odds of having IBD in those consuming at > Median monthly rate) | | | | | | | | | | | | | |
| ^i^Food items appear in more than one food groups: Pizza, Ice cream, Cereal | | | | | | | | | | | | | |
| *Statistically significant; Below the significance level of 0.05 | | | | | | | | | | | | | |
