# Supplementary material for: Examination of food consumption in United States adults and the prevalence of inflammatory bowel disease using National Health Interview Survey 2015
Source: PLoS One. 2020 Apr 23;15(4):e0232157. doi: 10.1371/journal.pone.0232157 (PMC7179926; doi:10.1371/journal.pone.0232157)
Supplement: S10 Table — (DOCX) [file pone.0232157.s010.docx]

| **Supplemental Table 10.1 Association (Odds^h^) of IBD and increasing consumption frequency in subpopulation with > or ≤ average (Median) food intake (Unadjusted), NHIS 2015^a,b^** | | | | | | | |
| --- | --- | --- | --- | --- | --- | --- | --- |
|  |  | Weighted, Unadjusted | | | Weighted, Unadjusted | | |
|  |  | Consumption Rate > Median | | | Consumption Rate ≤ Median | | |
| Food groups^c^ | Food items | OR | p-value | 95% CI | OR | p-value | 95% CI |
| Whole wheat grains | Popcorn | 0.97 | 0.110 | (0.9393 - 1.0064) | 0.62 | 0.014* | (0.4290 - 0.9087) |
|  | Cereal (hot or cold)^i^ | 1.00 | 0.535 | (0.9951 - 1.0095) | 1.08 | 0.107 | (0.9828 - 0.1954) |
|  | Brown rice | 1.00 | 0.768 | (0.9789 - 1.0159) | 0.96 | 0.902 | (0.5331 - 1.7420) |
|  | Whole grain bread | 0.99 | 0.225 | (0.9741 - 1.0623) | 0.91 | 0.002* | (0.8606 - 0.9679) |
| Fruits and vegetables | Fries | 1.01 | 0.380 | (0.9938 - 1.0164) | 1.19 | 0.006* | (1.0511 - 1.3433) |
|  | Salad (green leafy, lettuce) | 1.00 | 0.876 | (0.9891 - 1.0129) | 0.97 | 0.127 | (0.9413 - 1.0076) |
|  | Fruit juices (100% pure fruit juice) | 1.00 | 0.636 | (0.9952 - 1.0079) | 1.07 | 0.424 | (0.9026 - 1.2753) |
|  | Vegetables^d^ | 0.98 | 0.010* | (0.9621 - 0.9948) | 1.01 | 0.683 | (0.9782 - 1.0343) |
|  | Potato (non-fried) | 1.01 | 0.045* | (1.0002 - 1.0206) | 1.09 | 0.329 | (0.9207 - 1.2786) |
|  | Pizza (frozen, fast food, homemade)^i^ | 0.98 | 0.528 | (0.9344 - 1.0355) | 1.02 | 0.874 | (0.8160 - 1.2699) |
|  | Fruits (fresh, frozen, canned) | 1.00 | 0.508 | (0.9896 - 1.0052) | 1.00 | 0.745 | (0.9793 - 1.0296) |
|  | Tomato sauce | 1.01 | 0.484 | (0.9878 - 1.0263) | 0.99 | 0.873 | (0.8189 - 1.1849) |
|  | Salsa (made with tomatoes) | 0.98 | 0.122 | (0.9602 - 1.0048) | 0.87 | 0.224 | (0.6925 - 1.0904) |
|  | Beans | 1.00 | 0.564 | (0.9849 - 1.0083) | 1.04 | 0.599 | (0.8969 - 1.2072) |
| Dairy | Milk (cow milk, any type) | 1.00 | 0.896 | (0.9894 - 1.0094) | 0.99 | 0.749 | (0.9520 - 1.0361) |
|  | Cheese (excludes cheese on pizza) | 0.97 | 0.454 | (0.9900 - 1.0045) | 1.01 | 0.508 | (0.9758 - 1.0506) |
|  | Pizza (frozen, fast food, homemade)^i^ | 0.98 | 0.528 | (0.9344 - 1.0355) | 1.02 | 0.874 | (0.8160 - 1.2699) |
|  | Ice cream (frozen desserts)^i^ | 1.01 | 0.035* | (1.0007 - 1.0187) | 0.87 | 0.299 | (0.6747 - 1.1292) |
| Meat | Processed meat | 1.00 | 0.715 | (0.9839 - 1.0112) | 1.14 | 0.118 | (0.9670 - 1.3440) |
|  | Red meat | 1.00 | 0.478 | (0.9822 - 1.0085) | 1.03 | 0.322 | (0.9750 - 1.0796) |
| Sweetened food/drinks^e^ | Cereal (hot or cold)^i^ | 1.00 | 0.535 | (0.9951 - 1.0095) | 1.08 | 0.107 | (0.9828 - 0.1954) |
|  | Cookies (i.e. cake, pies, brownies) | 1.00 | 0.506 | (0.9925 - 1.0154) | 0.98 | 0.838 | (0.8133 - 1.1827) |
|  | Donut (i.e. Danish, pastries, muffins) | 1.01 | 0.071 | (0.9991 - 1.0225) | 1.37 | 0.107 | (0.9338 - 2.0131) |
|  | Coffee or tea (sugar or honey added) | 1.00 | 0.032* | (1.0004 - 1.0082) | 1.05 | 0.696 | (0.8327 - 1.3151) |
|  | Fruit drinks (sweetened with sugar) | 1.00 | 0.500 | (0.9808 - 1.0095) | n.a | n.a | n.a |
|  | Candy (i.e. chocolates) | 1.01 | 0.153 | (0.9981 - 1.0124) | 1.00 | 0.974 | (0.8445 - 1.1910) |
|  | Sports and energy drinks | 0.99 | 0.281 | (0.9727 - 1.0081) | n.a | n.a | n.a |
|  | Regular soda or pop | 1.01 | <0.001* | (1.0038 - 1.0111) | 1.05 | 0.748 | (0.7884 - 1.3921) |
|  | Ice cream (frozen desserts)^i^ | 1.01 | 0.035 | (1.0007 - 1.0187) | 0.87 | 0.299 | (0.6747 - 1.1292) |
|  |  |  |  |  |  |  |  |
|  |  |  |  |  |  |  |  |
|  |  |  |  |  |  |  |  |
| ^a^Weighted using sample weight [wtfa_sa]. Logistic regression with IBD as outcome; Data source: Sample Adult Cancer file from 2015 NHIS Data release source (https://www.cdc.gov/nchs/nhis/nhis_2015_data_release.htm) | | | | | | | |
| ^b^Additional details in survey questions can be found in NHIS 2015 Data release website: ftp://ftp.cdc.gov/pub/Health_Statistics/NCHS/Dataset_Documentation/NHIS/2015/cancerxx_layout.pdf | | | | | | | |
| ^c^Food groups are based on the relationship previously established according the dietary guidelines. Details can be found on https://epi.grants.cancer.gov/nhanes/dietscreen/relationship.html. | | | | | | | |
| ^d^Vegetables other than lettuce salads, potatoes, cooked beans in which participant already answered to in previous questions. | | | | | | | |
| ^e^Food items in this group excludes artificially sweetened or sugar-free kinds | | | | | | | |
| ^f^Each food item adjusted for demographic factors: Age, race, poverty status, sex, ethnicity, region | | | | | | | |
| ^g^Each food item adjusted for lifestyle factors: Smoking, alcohol user status, alcohol consumption rate, BMI | | | | | | | |
| ^h^Odds of having IBD with every unit increase in consumption of respective food item in the subgroup consuming either > Median or ≤Median | | | | | | | |
| ^i^Food items appear in more than one food groups: Pizza, Ice cream, Cereal | | | | | | | |
| n.a: The median for these diet items are 0, or none. Equivalent to having never consumed in past month. | | | | | | | |
| *Statistically significant; Below the significance level of 0.05 | | | | | | | |
|  |  |  |  |  |  |  |  |

| **Supplemental Table 10.2 Association (Odds^h^) of IBD and increasing consumption frequency in subpopulation with > or ≤ average (Median) food intake (Adjusted for demography), NHIS 2015^a,b^** | | | | | | | |
| --- | --- | --- | --- | --- | --- | --- | --- |
|  |  | Weighted, Adjusted for Demography | | | Weighted, Adjusted for Demography | | |
|  |  | **Consumption Rate > Median** | | | **Consumption Rate ≤ Median** | | |
| Food groups^c^ | Food items | OR | p-value | 95% CI | OR | p-value | 95% CI |
| Whole wheat grains | Popcorn | 0.97 | 0.098 | (0.9381 - 1.0055) | 0.66 | 0.030* | (0.4497 - 0.9591) |
|  | Cereal (hot or cold)^i^ | 1.00 | 0.700 | (0.9939 - 1.0091) | 1.08 | 0.120 | (0.9803 - 1.1870) |
|  | Brown rice | 1.00 | 0.635 | (0.9877 - 1.0204) | 0.99 | 0.969 | (0.5401 - 1.8075) |
|  | Whole grain bread | 0.99 | 0.168 | (0.9689 - 1.0055) | 0.93 | 0.012* | (0.8724 - 0.9830) |
| Fruits and vegetables | Fries | 1.01 | 0.183 | (0.9969 - 1.0164) | 1.23 | 0.001* | (1.0923 - 1.3865) |
|  | Salad (green leafy, lettuce) | 1.00 | 0.874 | (0.9890 - 1.0131) | 0.97 | 0.083 | (0.9363 - 1.0041) |
|  | Fruit juices (100% pure fruit juice) | 1.00 | 0.813 | (0.9938 - 1.0079) | 1.11 | 0.242 | (0.9327 - 1.3164) |
|  | Vegetables^d^ | 0.98 | 0.011* | (0.9622 - 0.9950) | 1.00 | 0.850 | (0.9738 - 1.0327) |
|  | Potato (non-fried) | 1.01 | 0.179 | (0.9965 - 1.0189) | 1.05 | 0.537 | (0.8923 - 1.2438) |
|  | Pizza (frozen, fast food, homemade)^i^ | 0.99 | 0.765 | (0.9440 - 1.0433) | 1.08 | 0.498 | (0.8593 - 1.3651) |
|  | Fruits (fresh, frozen, canned) | 1.00 | 0.505 | (0.9895 - 1.0052) | 1.01 | 0.603 | (0.9821 - 1.0316) |
|  | Tomato sauce | 1.01 | 0.412 | (0.9887 - 1.0280) | 0.99 | 0.887 | (0.8090 - 1.2014) |
|  | Salsa (made with tomatoes) | 0.99 | 0.297 | (0.9648 - 1.0111) | 0.93 | 0.544 | (0.7244 - 1.1857) |
|  | Beans | 1.00 | 0.906 | (0.9902 - 1.0112) | 1.02 | 0.764 | (0.8824 - 1.1857) |
| Dairy | Milk (cow milk, any type) | 1.00 | 0.868 | (0.9893 - 1.0091) | 1.00 | 0.817 | (0.9545 - 1.0375) |
|  | Cheese (excludes cheese on pizza) | 1.00 | 0.610 | (0.9913 - 1.0052) | 1.02 | 0.326 | (0.9805 - 1.0608) |
|  | Pizza (frozen, fast food, homemade)^i^ | 0.99 | 0.765 | (0.9440 - 1.0433) | 1.08 | 0.498 | (0.8593 - 1.3651) |
|  | Ice cream (frozen desserts)^i^ | 1.01 | 0.200 | (0.9961 - 1.0190) | 0.90 | 0.391 | (0.6950 - 1.1536) |
| Meat | Processed meat | 1.00 | 0.814 | (0.9857 - 1.0114) | 1.13 | 0.154 | (0.9556 - 1.3307) |
|  | Red meat | 1.00 | 0.468 | (0.9826 - 1.0081) | 1.03 | 0.263 | (0.9770 - 1.0796) |
| Sweetened food/drinks^e^ | Cereal (hot or cold)^i^ | 1.00 | 0.700 | (0.9939 - 1.0091) | 1.08 | 0.120 | (0.9803 - 1.1870) |
|  | Cookies (i.e. cake, pies, brownies) | 1.00 | 0.889 | (0.9871 - 1.0151) | 1.00 | 0.982 | (0.8280 - 1.2027) |
|  | Donut (i.e. Danish, pastries, muffins) | 1.01 | 0.082 | (0.9987 - 1.0226) | 1.47 | 0.053 | (0.9953 - 2.1649) |
|  | Coffee or tea (sugar or honey added) | 1.00 | 0.159 | (0.9989 - 1.0068) | 1.11 | 0.350 | (0.8872 - 1.4004) |
|  | Fruit drinks (sweetened with sugar) | 1.00 | 0.524 | (0.9813 - 1.0097) | n.a | n.a | n.a |
|  | Candy (i.e. chocolates) | 1.00 | 0.297 | (0.9963 - 1.0121) | 1.01 | 0.865 | (0.8566 - 1.2020) |
|  | Sports and energy drinks | 0.99 | 0.353 | (0.9755 - 1.0089) | n.a | n.a | n.a |
|  | Regular soda or pop | 1.01 | <0.001* | (1.0035 - 1.0108) | 1.10 | 0.501 | (0.8270 - 1.4736) |
|  | Ice cream (frozen desserts)^i^ | 1.01 | 0.200 | (0.9961 - 1.0190) | 0.90 | 0.391 | (0.6950 - 1.1536) |
|  |  |  |  |  |  |  |  |
|  |  |  |  |  |  |  |  |
|  |  |  |  |  |  |  |  |
|  |  |  |  |  |  |  |  |
| ^a^Weighted using sample weight [wtfa_sa]. Logistic regression with IBD as outcome; Data source: Sample Adult Cancer file from 2015 NHIS Data release source (https://www.cdc.gov/nchs/nhis/nhis_2015_data_release.htm) | | | | | | | |
| ^b^Additional details in survey questions can be found in NHIS 2015 Data release website: ftp://ftp.cdc.gov/pub/Health_Statistics/NCHS/Dataset_Documentation/NHIS/2015/cancerxx_layout.pdf | | | | | | | |
| ^c^Food groups are based on the relationship previously established according the dietary guidelines. Details can be found on https://epi.grants.cancer.gov/nhanes/dietscreen/relationship.html. | | | | | | | |
| ^d^Vegetables other than lettuce salads, potatoes, cooked beans in which participant already answered to in previous questions. | | | | | | | |
| ^e^Food items in this group excludes artificially sweetened or sugar-free kinds | | | | | | | |
| ^f^Each food item adjusted for demographic factors: Age, race, poverty status, sex, ethnicity, region | | | | | | | |
| ^g^Each food item adjusted for lifestyle factors: Smoking, alcohol user status, alcohol consumption rate, BMI | | | | | | | |
| ^h^Odds of having IBD with every unit increase in consumption of respective food item in the subgroup consuming either > Median or ≤Median | | | | | | | |
| ^i^Food items appear in more than one food groups: Pizza, Ice cream, Cereal | | | | | | | |
| n.a: The median for these diet items are 0, or none. Equivalent to having never consumed in past month. | | | | | | | |
| *Statistically significant; Below the significance level of 0.05 | | | | | | | |

| **Supplemental Table 10.3 Association (Odds^h^) of IBD and increasing consumption frequency in subpopulation with > or ≤ average (Median) food intake (Adjusted for lifestyle), NHIS 2015^a,b^** | | | | | | | |
| --- | --- | --- | --- | --- | --- | --- | --- |
|  |  | Weighted, Adjusted for Lifestyle | | | Weighted, Adjusted for Lifestyle | | |
|  |  | **Consumption Rate > Median** | | | **Consumption Rate ≤ Median** | | |
| Food groups^c^ | Food items | OR | p-value | 95% CI | OR | p-value | 95% CI |
| Whole wheat grains | Popcorn | 0.97 | 0.146 | (0.9414 - 1.0090) | 0.64 | 0.021* | (0.4437 - 0.9344) |
|  | Cereal (hot or cold)^i^ | 1.00 | 0.499 | (0.9953 - 1.0097) | 1.08 | 0.105 | (0.9832 - 1.1955) |
|  | Brown rice | 1.00 | 0.915 | (0.9813 - 1.0170) | 0.99 | 0.972 | (0.5442 - 1.7991) |
|  | Whole grain bread | 0.99 | 0.174 | (0.9705 - 1.0055) | 0.90 | 0.001* | (0.8500 - 0.9592) |
| Fruits and vegetables | Fries | 1.01 | 0.374 | (0.9939 - 1.0164) | 1.19 | 0.006* | (1.0518 - 1.3530) |
|  | Salad (green leafy, lettuce) | 1.00 | 0.813 | (0.9897 - 0.1013) | 0.97 | 0.136 | (0.9406 - 1.0084) |
|  | Fruit juices (100% pure fruit juice) | 1.00 | 0.560 | (0.9956 - 1.0082) | 1.08 | 0.402 | (0.9047 - 1.2824) |
|  | Vegetables^d^ | 0.98 | 0.011* | (0.9626 - 0.9950) | 1.01 | 0.504 | (0.9811 - 1.0395) |
|  | Potato (non-fried) | 1.01 | 0.043* | (1.0003 - 1.0207) | 1.11 | 0.247 | (0.9314 - 1.3166) |
|  | Pizza (frozen, fast food, homemade)^i^ | 0.98 | 0.517 | (0.9336 - 1.0353) | 1.06 | 0.628 | (0.8421 - 1.3291) |
|  | Fruits (fresh, frozen, canned) | 1.00 | 0.558 | (0.9899 - 1.0055) | 1.01 | 0.654 | (0.9801 - 1.0324) |
|  | Tomato sauce | 1.01 | 0.535 | (0.9871 - 1.0253) | 0.98 | 0.847 | (0.8109 - 1.1879) |
|  | Salsa (made with tomatoes) | 0.98 | 0.141 | (0.9603 - 1.0058) | 0.89 | 0.340 | (0.7075 - 1.1272) |
|  | Beans | 1.00 | 0.587 | (0.9852 - 1.0085) | 1.06 | 0.463 | (0.9081 - 1.2355) |
| Dairy | Milk (cow milk, any type) | 1.00 | 0.894 | (0.9890 - 1.0097) | 1.00 | 0.849 | (0.9541 - 1.0395) |
|  | Cheese (excludes cheese on pizza) | 1.00 | 0.464 | (0.9903 - 1.0045) | 1.01 | 0.543 | (0.9738 - 1.0517) |
|  | Pizza (frozen, fast food, homemade)^i^ | 0.98 | 0.517 | (0.9336 - 1.0353) | 1.06 | 0.628 | (0.8421 - 1.3291) |
|  | Ice cream (frozen desserts)^i^ | 1.01 | 0.030* | (1.0009 - 1.0186) | 0.88 | 0.323 | (0.6742 - 1.1393) |
| Meat | Processed meat | 1.00 | 0.728 | (0.9840 - 1.0114) | 1.15 | 0.119 | (0.9650 - 1.3669) |
|  | Red meat | 0.99 | 0.448 | (0.9816 - 1.0083) | 1.03 | 0.221 | (0.9808 - 1.0872) |
| Sweetened food/drinks^e^ | Cereal (hot or cold)^i^ | 1.00 | 0.499 | (0.9953 - 1.0097) | 1.08 | 0.105 | (0.9832 - 1.1955) |
|  | Cookies (i.e. cake, pies, brownies) | 1.00 | 0.411 | (0.9937 - 1.0156) | 0.98 | 0.850 | (0.8107 - 1.1889) |
|  | Donut (i.e. Danish, pastries, muffins) | 1.01 | 0.044* | (1.0003 - 1.0226) | 1.33 | 0.155 | (0.8965 - 1.9853) |
|  | Coffee or tea (sugar or honey added) | 1.00 | 0.038* | (1.0002 - 1.0083) | 1.05 | 0.650 | (0.8410 - 1.3196) |
|  | Fruit drinks (sweetened with sugar) | 0.99 | 0.438 | (0.9800 - 1.0088) | n.a | n.a | n.a |
|  | Candy (i.e. chocolates) | 1.01 | 0.117 | (0.9986 - 1.0124) | 1.02 | 0.870 | (0.8490 - 1.2135) |
|  | Sports and energy drinks | 0.99 | 0.325 | (0.9746 - 1.0086) | n.a | n.a | n.a |
|  | Regular soda or pop | 1.01 | <0.001* | (1.0037 - 1.0111) | 1.07 | 0.666 | (0.7977 - 1.4233) |
|  | Ice cream (frozen desserts)^i^ | 1.01 | 0.030* | (1.0009 - 1.0186) | 0.88 | 0.323 | (0.6742 - 1.1393) |
|  |  |  |  |  |  |  |  |
|  |  |  |  |  |  |  |  |
|  |  |  |  |  |  |  |  |
|  |  |  |  |  |  |  |  |
|  |  |  |  |  |  |  |  |
| ^a^Weighted using sample weight [wtfa_sa]. Logistic regression with IBD as outcome; Data source: Sample Adult Cancer file from 2015 NHIS Data release source (https://www.cdc.gov/nchs/nhis/nhis_2015_data_release.htm) | | | | | | | |
| ^b^Additional details in survey questions can be found in NHIS 2015 Data release website: ftp://ftp.cdc.gov/pub/Health_Statistics/NCHS/Dataset_Documentation/NHIS/2015/cancerxx_layout.pdf | | | | | | | |
| ^c^Food groups are based on the relationship previously established according the dietary guidelines. Details can be found on https://epi.grants.cancer.gov/nhanes/dietscreen/relationship.html. | | | | | | | |
| ^d^Vegetables other than lettuce salads, potatoes, cooked beans in which participant already answered to in previous questions. | | | | | | | |
| ^e^Food items in this group excludes artificially sweetened or sugar-free kinds | | | | | | | |
| ^f^Each food item adjusted for demographic factors: Age, race, poverty status, sex, ethnicity, region | | | | | | | |
| ^g^Each food item adjusted for lifestyle factors: Smoking, alcohol user status, alcohol consumption rate, BMI | | | | | | | |
| ^h^Odds of having IBD with every unit increase in consumption of respective food item in the subgroup consuming either > Median or ≤Median | | | | | | | |
| ^i^Food items appear in more than one food groups: Pizza, Ice cream, Cereal | | | | | | | |
| n.a: The median for these diet items are 0, or none. Equivalent to having never consumed in past month. | | | | | | | |
| *Statistically significant; Below the significance level of 0.05 | | | | | | | |
